# Towards precision epitopes based vaccine against *Enterococcus faecalis* by integrating vaccinomics, reverse vaccinology and biophysics approaches

**DOI:** 10.1016/j.bbrep.2025.102082

**Published:** 2025-06-10

**Authors:** Asad Ullah, Sadiq Azam, Sajjad Ahmad, Ibrar Khan, Dalia M. Alammari, Sumra Wajid Abassi, Dong-Qing Wei, Fahad M. Alshabrmi, Mohammad Abdullah Aljasir, Eid A. Alatawi

**Affiliations:** a Centre of Biotechnology and Microbiology, University of Peshawar, 25120, Peshawar, Pakistan; b Department of Health and Biological Sciences, Abasyn University, 25000, Peshawar, Pakistan; c Zhongjing Research and Industrialization Institute of Chinese Medicine, Zhongguancun Scientific Park, Nayang, PR China; d Department of Microbiology and Immunology, Faculty of Medicine Program, Ibn Sina National College of Medical Studies, Jeddah, Saudi Arabia; e Department of Biological Sciences, National University of Medical Sciences, Pakistan; f Department of Medical Laboratories, College of Applied Medical Sciences, Qassim University, Buraydah, 51452, Saudi Arabia; g Department of Medical Laboratory Technology, Faculty of Applied Medical Sciences, University of Tabuk, Tabuk, 71491, Saudi Arabia

**Keywords:** Antibiotics resistance, *Enterococcus faecalis*, Multi-epitopes-based vaccine, Reverse vaccinology, ImmunoInformatics, Molecular docking simulation, C-immune simulation

## Abstract

*Enterococcus faecalis* is a Gram-positive bacterium and recognized as an etiological agent of different nosocomial infections. *E. faecalis* has developed resistance to several antibiotics. No licensed vaccine is available to prevent *E. faecalis* infections. A multi-epitopes-based vaccine construct may provide effective vaccine design foundation. In this study, an integrated bioinformatic approach was applied to design of a multi-epitopes-based vaccine construct against *E. faecalis*. In subtractive proteomics analysis, 10 proteins were prioritized as potential vaccine candidates based on several literature reported vaccine candidacy parameters. In immunoinformatics analysis, only two proteins (glucosaminidase domain-containing protein and serine protease) were found as promising vaccine targets. Both proteins were then subjected to epitopes mapping for screening of broad-spectrum antigenic epitopes. The predicted epitopes were further refined based on immunoinformatics filters and only six epitopes; DTSDHQKNNV, GMKKRKARY, SVFDESMALR, NLNQRIEKR, NVDKKIEEK, and TTTPSTDNSA were found as non-allergic, antigenic, water-soluble, non-toxic and DRB∗0101 good binders. The selected epitopes were fused via GPGPG linkers and additionally linked to an adjuvant molecule through EAAAK linkers to increase the immunogenicity and antigenicity of the vaccine construct. The net interactions energy of the vaccine and receptors was evaluated through molecular docking analysis, which predicted −833.0 kcal/mol and −1001.6 kcal/mol of binding energy for MHC-I and MHC-II, respectively. The values predict effective vaccine construct binding with host immune cell receptors and triggering of innate and adaptive immune responses. The dynamic behavior of the docked complexes was examined using molecular dynamics (MD) simulation technique on a time scale of 500 ns. The MD revealed minimal intermolecular conformational deviations and exposed presentation of the vaccine epitopes for immune cells recognition and processing. For MHC-I-Vaccine complex, the mean RMSD was found as 2.78 Å while MHC–II–Vaccine complex showed a mean RMSD value of 13.17 Å. The C-immune simulation predicted the formation of high titer humoral and cellular immunological responses against the vaccine antigen. The predicted IgG and IgM titer found against the antigen was 600000–650000 counts per milliliter. The interferon-gamma (IFN-γ) was predicted to be stimulated at 430000 ng per milliliter. Simulation trajectories based MMGB/PBSA binding energy was estimated as < −250 kcal/mol for vaccine-MHC complexes, illustrating formation of robust interactions between the vaccine and MHC receptors. The study outcomes predicted the viability of the proposed epitope-based vaccine construct as a promising therapeutic approach for *E. faecalis* infections prevention, however, experimental confirmation is required.

## Highlights

• glucosaminidase domain-containing protein and serine protease were found as promising vaccine targets.
• Only six epitopeswere found as non-allergic, antigenic, water-soluble, and non-toxic.
• The net interactions energy of the vaccine and receptors was evaluated through molecular docking analysis.
• For MHC-I-Vaccine complex, the mean RMSD was found as 2.78 Å. MHC–II–Vaccine complex showed a mean RMSD value of 13.17 Å.

## 1 Introduction

Antibiotic resistance (AR) by bacterial pathogens has been described as one of the biggest global healthcare challenges of the modern era. AR poses a serious risk to public health and is evolving rapidly among bacterial, viral, and fungal infections [1]. Some Gram-negative and Gram-positive bacterial pathogens such as *Acinetobacter baumannii*, *Enterobacteriaceae*, and *Enterococcus* are more resistant and pathogenic and thus have limited available treatment options [2]. During organ transplantation and chemotherapy for cancer, the patients are more vulnerable to microbial infections due to the administration of immunosuppressive drugs. During such times, the widespread increase of AR in the healthcare system can cause nosocomial infections, which are difficult to treat. AR is a global health concern and needs tracking and control not only in human medication but also in animal farming, agricultural and fish farming. AR occurs when a microbial pathogen grows in the presence of antibiotic concentration that would normally kill or prevent microbial pathogens [3]. Bacterial develop AR by different mechanisms including genetic mutations, horizontal gene transfer, efflux pumps, and enzymatic degradation of drugs [4].The Enterococcus genus comprises Gram-negative bacteria responsible for myriad nosocomial infections such as infections of the bloodstream, urinary tract, surgical site, and organ transplant related infections [5].

Enterococci are highly adaptable species that can survive hostile environments. Among *enterococci*, two species (*E. faecalis* and *E. faecium*) are main etiological agents of hospital-associated infections. Both species have revealed intrinsic resistance to common antibiotics such as gentamicin, amikacin, tobramycin, neomycin, streptomycin and Trimethoprim/sulfamethoxazole (TMP/SMX) [6]. *E. faecalis* and *E. faecium* promptly acquire resistance to high level of antibiotics either through genetic mutation or by horizontal gene transfer [7]. *E. faecalis* is a Gram-positive commensal bacterium mostly present in the gastrointestinal tract of humans. *E. faecalis* is the third most commonly isolated pathogen from health care settings and causes different nosocomial infections such as endocarditis, bloodstream infection post-surgical and urinary tract infection (UTI). Due to misuse and overuse of antibiotics, *E. faecalis* has developed resistance to multiple antibiotics [8].Vaccination is an effective approach in eradicating bacterial infections. However, vaccine development for many bacterial pathogens is difficult due to several technical challenges [9] such as genetic variability of the pathogens, pathogenic pathways, complete understanding of host and pathogens association and safety [10].

Recent advancements in omics sciences played a crucial role in the development of vaccines. The growing use of genomic approaches also helped in understanding host-pathogen interactions and disclosing potent novel antigens not known before [11]. Multi-epitopes vaccines (MEVs) are novel vaccine development strategy that stimulate strong immune responses against multiple antigenic epitopes from pathogen core proteins. MEVs vaccines are capable of triggering both cellular and humoral immunity making them particularly effective against complex infections of viruses, parasites, fungi and bacteria [12,13]. Furthermore, MEVs have different key aspects, such as consisting of a combination of T-cell (MHC class I and II), helper T-cell (Th), and B-cell epitopes, all of which can provoke a high magnitude of protective immune responses [14].

Docking and simulation investigation are essential in silico pipelines in vaccine development. These approaches elucidate the interactions between vaccine candidates and host immune cells receptors for understanding effective immunization mechanisms [15]. The C-immune simulation analysis involves computational assisted methods to model and simulate host immune system dynamics against given vaccine antigen under different set of conditions. It combines machine learning and agent based algorithms for profiling immune responses and facilitate development of vaccine and therapeutic strategies [16].

This study aims to employ reverse vaccinology, immunoinformatics, and biophysics methodologies to make multi-epitopes vaccine targeting *E. faecalis*. Subsequent to vaccine construct design, C-immune simulation was conducted to predict the efficacy of the vaccine design in provoking immunological responses. Though significant progress has been made in computational vaccine designing, several critical research gaps still need to be addressed. Initially, comprehensive experimental research is necessary to evaluate the simulation of host defenses and their protective effectiveness across diverse numbers of populations [17]. Second, while bioinformatics tools and pipelines have significantly improved epitopes prediction and selection, consistent protocols for experimentally validating these predictions are lacking. Furthermore, the choice of adjuvant and delivery systems tailored specifically for multi-epitopes vaccine constructs remain underexplored, which may limit the effectiveness of these vaccines. Lastly, a deeper understanding of immune system tolerance mechanisms and probable adverse effects associated with multi-epitopes vaccines is critical to ensure their safety level and effectiveness in clinical trial phases [18]. Addressing these research gaps is vital for effectively transitioning of multi epitopes-based vaccine candidates from the laboratory to clinical trial phases.

## 2 Materials & methods

**Fig. 1:**
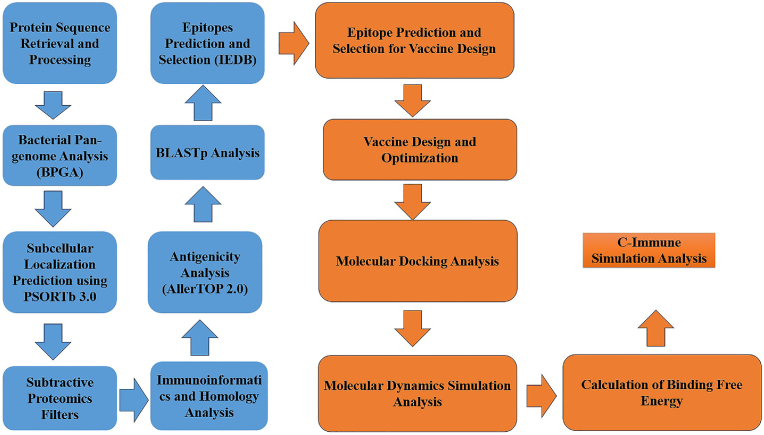
This flow diagram demonstrates the in-silico pipeline for multi-epitopes vaccine design starting from protein sequence retrieval to be used in subtractive proteomics filtering to identify potential vaccine targets. The major phases comprise epitopes prediction, antigenicity assessment and subcellular localization analysis. Afterward, the vaccine construction and processing, molecular docking, molecular dynamics simulation and binding free energy calculation were performed. The last phase contains C-ImmSim host immune simulation analysis to evaluate the host immune responses ensuring the effectiveness of designed vaccine construct.

The research workflow depicted in Fig. 1 was utilized to perform all phases for vaccine construct designing against *E. faecalis.*

### 2.1 Protein sequence retrieval and processing

The protein sequences of the available 20 fully sequenced strains of *E. faecalis* were obtained from the NCBI database [19]. The sequences were processed for core proteins sequence prediction through bacterial pan genome analysis (BPGA) [20]. Using BPGA, core proteins sequences, pan core plot histogram, and genes plot were generated. Next, the core sequences were subsequently subjected to subcellular localization prediction using PSORTb 3.0 [21]. PSORTb 3.0 is a computational tool for predicting subcellular localization of proteins. The prediction of surface proteins in bacterial pathogens is particularly significant since these proteins may serve as major targets for vaccines. The subcellular localization of a protein is determined by various characteristics in its fundamental structure including the signal peptide or membrane-spanning alpha-helices [22]. In PSORTb analysis, only surface associated proteins were shortlisted and cytoplasmic membrane proteins were discarded. After surface localization analysis, virulent proteins sequences were analyzed using Virulence Factor Database (VFDB). In VFDB analysis, proteins were shortlisted that revealed a bit score of >100 and sequence identity of >30 % [23].The virulent proteins were additionally subjected to physicochemical properties analysis to select only those protein sequences that are physiochemically stable and easy to use in experimental studies likely pilot and clinical studies trials. Physicochemical properties analysis was done using the ProtParam tool [24]. This tool permits different computational parameters including instability index, molecular weight, etc. The key parameters assessed during this analysis were molecular weight and instability index. The shortlisted proteins revealed a <110 kDa (kDa) molecular weight. Molecular weight calculating analysis was considered essential from the purification and development perspective. In instability index analysis, those proteins were selected with predicted value < 40. The instability calculator mainly determines the instability value by predicting the occurrence of specific dipeptides that are absent in vivo and unstable but found in stable protein [25].

### 2.2 Immunoinformatics and homology analysis

In immunoinformatic analysis, the selected core, virulent, physiochemically stable proteins were used in antigenicity analysis. Antigenicity testing was accomplished by using VaxiJen 2.0 server [26]. In VaxiJen 2.0 server, bacteria were selected as the target pathogen and cut off value of 0.4 was used [27].Further, allergic proteins were removed through AllerTOP v2.1 allergen prediction tool [28]. To avoid autoimmunity and accidental growth inhibition of normal microbiota, a BLASTp analysis was done against Human (taxid: 9606) and *Lactobacillus* species including *L. rhamnosus* (taxid: 47715), *L. casei* (taxid: 1582), and *L. johnsonii* (taxid: 33959). In BLASTp analysis, sequence similarity >30 % and bit score >100 and 0.005 E-value were considered as thresholds. Next, a homology analysis against mouse proteome (tax id: 10088) was done to select proteins that are non-similar to *mus musculus* (taxid:10090). This analysis aimed to reduce autoimmune responses during an in vivo experimental study [29]. The filtered non-similar proteins were selected for epitopes prediction.

### 2.3 Prediction and selection of epitopes

In epitopes prediction and selection phase, first linear B cell epitopes were predicted followed by T cell epitopes. For linear B cell epitopes, Bepipred Linear Epitope Prediction 2.0 with threshold value of 0.5 at Immune Epitope Database and Analysis Resource (IEDB) was used [30]. Both MHC class I and MHC class II were then anticipated in the T cell epitopes prediction using predicted B cell epitopes [31]. The anticipated epitopes were prioritized in accordance with their lowest percentile score. Additionally, all selected epitopes underwent antigenic evaluation, allergenicity check, toxicity check, solubility and Mhcpred 2.0 analysis using VaxiJen [32], ToxinPred [33], peptide calculator, and MHCPred 2.0, respectively. In Mhcpred 2.0 analysis, only those epitopes were selected having IC50 value < 100 nM for DRB∗0101 allel. In the epitopes selection phase, only antigenic, non-allergens, good water soluble, non-toxic and DRB∗0101 binders were selected for multi-epitopes vaccine construction [34]. The identified and chosen epitopes were subjected to population coverage analysis using IEDB database [35].

### 2.4 Vaccine construction and processing

A significant problem related to peptide-based vaccinations is lack of generating strong immune responses. This limitation can be reduced by selecting and combining dominant antigenic determinants (epitopes) and construction of multi-epitopes based vaccine design using appropriate linkers and adjuvants [36]. In vaccine construct design, filtered and final selected epitopes were joint utilizing GPGPG linker. Further, the epitopes peptide was connected to cholera toxin B subunit (CTB) by an another EAAAK linker. A linear sequence of vaccine was subjected to structure prediction and immunoinformatics analysis [37]. Tertiary structure was predicted using an in-silico SCRATCH bioinformatics tool [38]. Subsequently, the loops in the predicted structure were modeled using Galaxy Loop refine [39]. An in-silico Design 2.0 webserver was employed for the disulfide engineering analysis of the modeled vaccine [40]. To optimize different codon usage in *E. coli* to the original host, the vaccine construct was reverse transcribed. The Java Codon Adaptation Tool (JCat) was employed to enhance the cloned sequence expression level within the expression system [40]. The cloned sequence expression intensity was assessed based on the percentage of GC content and the codon adaptation index (CAI) value. The optimal cut-off value of 1 for CAI was considered ideal. The preferred GC content was maintained between 30 % and 70 % as this range correlates with enhanced transcriptional and translational efficiency. The reverse transcribed sequence of vaccine construct was then cloned into the pET-28a (+) vector by using in silico SnapGene tool [41].

### 2.5 Molecular docking analysis

Molecular docking study of the vaccine and immune cell receptors involves multiple crucial steps that have been followed. Firstly, three-dimensional structures of the target receptors (MHC-I and MHC-II with PDB-ID of 1I1Y and 1KG0, respectively) were obtained from the Protein Data Bank (PDB) [42]. Additionally, the vaccine construct was saved in PDB format because ClusPro only accepts PDB format [43]. The ClusPro web server was accessed for docking purpose keeping the parameters as default [44,45]. The ClusPro server executes rigid body docking by sampling billions of conformations of the vaccine relative to the MHC-I and MHC-II. This server rotates the ligand approximately 70,000 times and translates it into three dimensions (x, y, z) for each rotation. The docking results were analyzed based on cluster size and binding energy scores. Furthermore, the docked complexes were visualized in the UCSF Chimera v1.19 [46] to examine for structure stability and assess the binding interactions [27].

### 2.6 Molecular dynamic simulation analysis

Molecular dynamics (MD) simulation is critical for understanding the movement behavior of docked molecules. The top-docked complexes were examined in 500 ns (ns) production run [47]. This analysis was carried out across multiple key phases: system preparation, pre-processing, production and simulation plots analysis. The first three phases were achieved through AMBER 2022 [48]. During the system preparation phase, antechamber package was used to make complexes libraries and parameters of docked complexes. Next, the Leap program was used for solvation of docked complexes in TIP3P solvation box. The box size was sized as 12 Å. The ff14SB was used for explanation of intermolecular interactions of docked molecules. The systems were neutralized through the incorporation of 25 sodium (Na+) counter ions [37].The subsequent preprocessing step focused on preparing the systems for the production run. The kinetic energy of the docked molecules was minimized as: minimization of hydrogen atoms (500 cycles), minimization of the water box (1000 cycles), minimization of all system atoms (1000 cycles with a restraint of 5 kcal/mol Å^2^ on Cα atoms), and minimization of non-heavy atoms (300 cycles with a restraint of 100 kcal/mol Å^2^). Afterward, the docked complexes were heated at a time step of 2 fs throughout 20 ps. Langevin dynamics was employed to regulate the temperature with the input gamma value configured at 1.0. SHAKE algorithm was used to constrain hydrogen bonds (Nosé, 1984). Subsequently, the docked complexes were equilibrated for 100 ps. The pressure equilibrium was obtained using an NPT ensemble, followed by an extended 50-ps simulation employing the same parameters. During the equilibrium phase, the docked complexes were allowed to reach equilibrium over a timescale of 1 ns [34]. In the production phase, molecular dynamics trajectories of 500 ns were generated. The Berendsen method was employed during the production phase. A cutoff value of 8.0 Å was set to differentiate nonbounded interactions [25]. The CPPTRAJ module was utilized to investigate the simulated trajectories of AMBER 2022. XMGrace v5.1 and VMD v1.93 were employed for the observing and interpretation of simulation trajectories [49].

### 2.7 Estimation of relative binding free energy

Binding free energy calculation is essential for evaluating the interaction of vaccine and receptor molecules. Two known methodologies, MM/GBSA and MM/PBSA methods, implemented in AMBER22 software, were applied to determine the binding free energy calculation [50]. The Generalized Born (GB) and Poisson Boltzmann (PB) models were chosen due to their less computational cost and adequate accuracy in estimating relative binding affinities. Furthermore, MMGB/SA mainly mixes molecular mechanics with the generalized born model to estimate free binding energy by accounting for both solvation effects and molecular interactions. This method is extensively used to analyze the docking stability, accuracy, and validation of computational findings. On the other hand, the MMPB/SA approach exploits the Poisson-Boltzmann equation to offer a detailed illustration of electrostatic interactions. The MMPB-SA approach is particularly valued when accurate electrostatic contributions are essential for understanding the binding capacity of vaccines with immune cell receptors [47].

The binding energy calculations of the dock complexes employed a dielectric constant of 1.0 (to measure electrostatic interactions between solute and solvent) and a solvent dielectric constant of 80.0, consistent with standard practice for aqueous systems. The free energy calculations were based on 500 snapshots (frames) extracted from the last 100 ns of the MD trajectory at regular intervals to confirm appropriate sampling of the conformational space. The entropy energy was omitted during this process as it is computationally expensive for such large complexes.

### 2.8 C-immune simulation analysis

Host immune simulation analysis is a critical stage in vaccine development to analyze the effectiveness of vaccine. C-immune simulation analysis was done using the C-ImmSim server. This tool focuses on simulating the host immune system to evaluate the efficacy of the designed multi-epitopes-based vaccine construct. The server was accessed at https://kraken.iac.rm.cnr.it/C-IMMSIM/index.php [51]. The linear sequence of vaccine was used as an input file and pasted into the C-ImmSim interface followed by setting all the simulation parameters including the number and duration of simulations, and type of immune response i.e. humoral and cellular immune responses. Afterward, selecting all the parameters, simulation was run and evaluate the immune responses over time. The interactions of B cell and T cell and others including cytokines and interleukins were noticed [52].

## 3 Results

### 3.1 Protein sequences retrieval and subtractive proteomic analysis

**Fig. 2:**
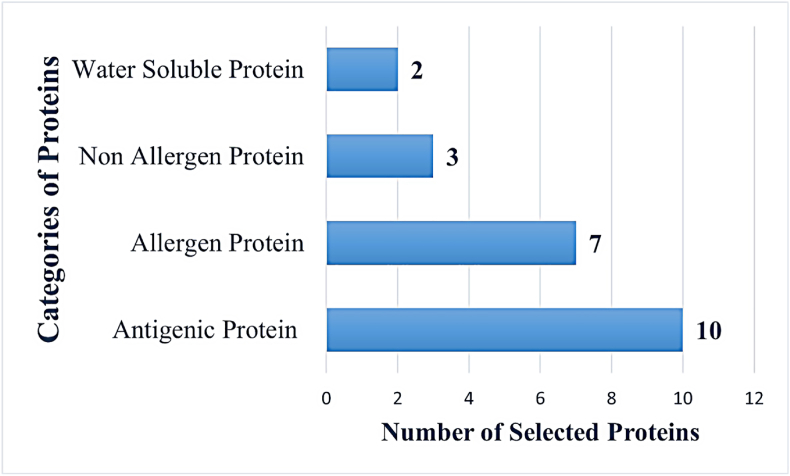
Subtractive proteomic analysis for prioritizing antigenic, non-allergic and water-soluble proteins. The number of proteins found in each step of this phase were as; antigenic (n = 10), allergen (n = 7), non-allergen (n = 3) and water soluble (n = 2).

On February 4, 2022, protein sequences from 20 fully sequenced strains of *E. faecalis* were retrieved from NCBI database. An investigation of the bacterial pan-genome predicted 37,720 essential core proteins. S-Fig. 1 depicts the core-pan plot, gene family distribution and gene families across the bacterial species showing a high number of unique and conserved genes. The anticipated core proteins underwent sub-cellular localization investigation revealing 33 proteins localized extracellularly. In the VFDB analysis, 18 proteins were classified as virulence factor. The virulence factors were subjected to physicochemical properties analysis which predicted 8 unstable and 10 stable proteins. The shortlisted proteins and their categories are presented in S-Fig. 2. In antigenicity analysis, all 10 shortlisted proteins were found as probable antigens. In the allergenicity assessment, seven proteins were found as probable allergens. The non-allergic proteins were further subjected to water solubility analysis. Only two proteins (glucosaminidase domain-containing protein and serine protease) were predicted as good water soluble. In BLASTp analysis, the selected two proteins demonstrated no hit against human, *mus musculus* and selected normal flora bacterial species. Fig. 2 depicts the numbers and categories of proteins that were selected and discarded during the subtractive proteomics analysis.

### 3.2 Epitopes prediction

**Fig. 3:**
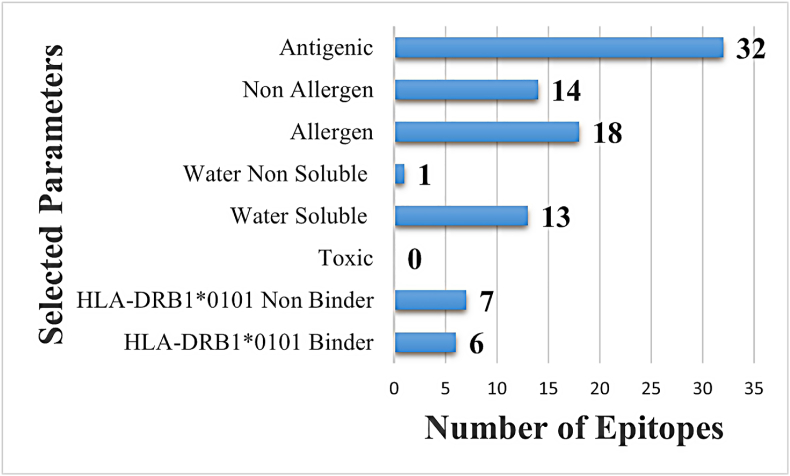
Immunoinformatics analysis for the selection of potential epitopes to be used in the construction of a multi-epitopes-based vaccine construct against *E. faecalis*. The immunoinformatics filters comprises antigenicity, allergenicity, solubility, toxicity, and HLA- DRB∗0101 binding ability. The epitopes filtered in different stages of this phase were; antigenic (n = 32), non-allergic (n = 14), and non-toxic (n = 0). Water soluble epitopes were 13 and 1 was poor water-soluble peptide. Also, seven HLA-DRB∗0101 non-binder and six epitopes were predicted HLA-DRB∗0101 good-binder.

Based on subtractive proteomics filters and immunoinformatics analysis, two proteins (glycosaminidase domain-containing protein and serine protease) were prioritized for epitope prediction. For B and T cell epitopes prediction, IEDB database was used. In this analysis, 13 B-cell epitopes of different lengths were forecasted from glucosaminidase domain-containing protein and 3 epitopes were predicted from serine protease. The anticipated B cell epitopes are tabulated in S-Table 1. Additionally, the B-cell epitopes graphs are shown in S-Fig. 3. The B-cell epitopes underwent additional analysis for the prediction of T-cell epitopes. In this step, both MHC-I and MHC-II epitopes were anticipated. The anticipated T-cell epitopes along with their least percentile scores are presented in S-Table 2.

### 3.3 Epitopes level immunoinformatics analysis

Further screening was applied to select epitopes for multi-epitopes vaccine construction. Different immunoinformatics filters were applied, including antigenicity, allergenicity assessment, water solubility, toxicity, and DRB∗0101 binding ability analysis. Only antigenic, non-allergens, water-soluble, non-toxic, and DRB∗0101 binder epitopes were selected for vaccine construct designing. In antigenicity analysis, only 32 epitopes were predicted as probable antigenic. The 18 epitopes were found as allergen epitopes whereas 14 were non-allergen. The allergic epitopes were removed and the non-allergen epitopes underwent water solubility analysis. This analysis predicted 13 epitopes as water soluble and 1 was predicted non-water-soluble. The water-soluble epitopes were subjected to toxicity analysis. In toxicity analysis, all the shortlisted epitopes were found to be non-toxic. Only 6 epitopes were further predicted as good DRB∗0101 binders and 7 epitopes were predicted as a DRB∗0101 non-binder. Fig. 3. shows the number and categories of selected and discarded epitopes. Subsequently, from the immunoinformatics analysis, only those epitopes that were antigenic, non-allergenic, highly water-soluble, non-toxic, and effective binders for DRB∗0101 were chosen for vaccine construction.

### 3.4 Analysis of population coverage for selected epitopes

**Fig. 4:**
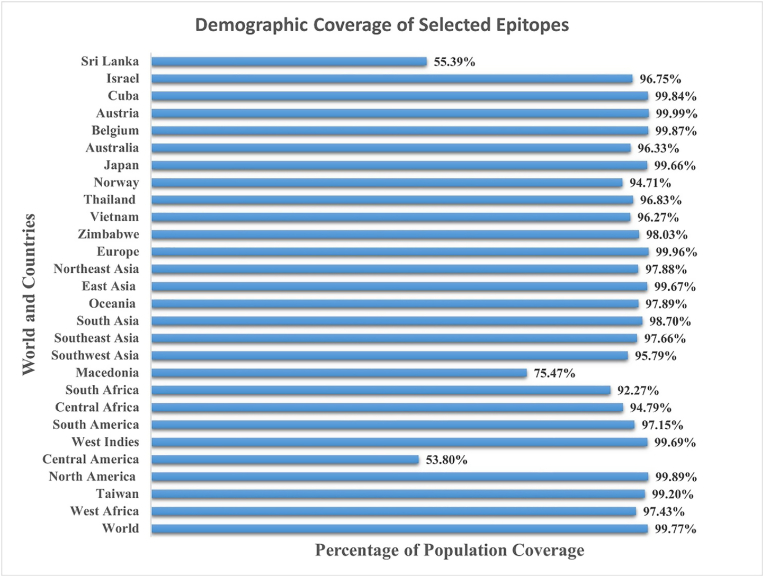
Population coverage analysis of selected epitopes for designing of multi-epitopes-based vaccine construct showing protective immune responses against the vaccine antigens due to their interactions with diverse human HLA alleles.

In population convergence analysis, the selected epitopes showed high HLA alleles coverage, which subsequently revealed population coverage more than 95 %, of the world's population due to the high prevalence of the choose set of HLA alleles in human populations. Notably, almost complete coverage is seen in North America (99.89 %), Europe (99.96 %), Cuba (99.84 %), and Austria (99.99 %). While Sri Lanka (55.39 %) and Central America (53.80 %) exhibited markedly lower coverage relative to the global average. This indicates that the chosen epitopes may be not suitable for certain groups probably because of genetic HLA variances. The international coverage is 99.77 % indicating that these epitopes are substantially covered in the worldwide population. The country-wise coverage of the vaccine is; South Africa (92.27 %), Central Africa (94.79 %), West Indies (99.69 %) and Oceania (97.89 %) exhibited notably high coverage. East Asia (99.67 %) and Northeast Asia (97.88 %) demonstrated robust HAL coverage. Zimbabwe (98.03 %) demonstrated better coverage than South Africa (92.27 %) and Central Africa (94.79 %). In Asia, nations like Japan (99.66 %) and Taiwan (99.20 %) have great coverage, whilst Sri Lanka presents a notable deviation with significantly lower vaccine coverage. North America (99.89 %) and the West Indies (99.69 %) have practically total coverage. Central America (53.80 %) demonstrates a markedly lower coverage percentage while European countries are predicted to be completely protected by the vaccine antigens. This analysis provides a rough estimate for vaccinologist about the possible HLA coverage of the proposed vaccine formulation ensuring that the selected epitopes effectively target a significant percentage of the worldwide population. Fig. 4 illustrates the demographic distribution of selected epitopes across different countries and regions. S-Table 3 depicts selected epitopes and HLA alleles. The vaccine epitopes showed binding to diverse HLA alleles, which show wide demographic coverage and are present in most of the world's population. Thus, it can be predicted that the proposed vaccine epitopes showed high percentage of population coverage, that is, 99.77 %.

### 3.5 Multi-epitopes vaccine designing

In the multi-epitopes vaccine designing phase, selected epitopes (Table 1) were linked together by GPGPG linkers and then connected to EAAAK linker for adjuvant addition. A linear sequence of vaccine was designed and evaluated for physicochemical characteristics.

**Table 1:** Immunoinformatics analysis of Selected epitopes according to percentile score, antigenicity, allergenicity, water solubility, toxicity and DRB∗0101 binding ability.

Selected epitopes	Percentile Score	Antigenicity Evaluation	Allergenicity Evaluation	Solubility in water	Toxicity	DRB∗0101 binding
DTSDHQKNNV	0.28	1.18	Non-allergens	Good water soluble	Non-toxic	7.26
GMKKRKARY	0.28	1.39	Non-allergens	Good water soluble	Non-toxic	46.03
SVFDESMALR	0.01	0.51	Non-allergens	Good water soluble	Non-toxic	29.31
NLNQRIEKR	0.09	1.70	Non-allergens	Good water soluble	Non-toxic	43.25
NVDKKIEEK	0.83	1.72	Non-allergens	Good water soluble	Non-toxic	74.13
TTTPSTDNSA	0.84	0.74	Non-allergens	Good water soluble	Non-toxic	8.15

### 3.6 Immunoinformatics and physiochemical properties analysis of vaccine construct

The antigenicity, allergenicity, water solubility and toxicity of the designed vaccine construct were assessed. The vaccine construct is probable antigen and capable of provoking an immune response. Additionally, the vaccine construct was found non-allergenic, good water-soluble and non-toxic. The vaccine candidate contains 211 amino acids, has a molecular weight of 22.862 kDa, and an instability index value of 30. Thus, the vaccine was considered as stable. S-Table 4 represents the findings of the immunoinformatics analysis and physicochemical characteristics of the proposed vaccine construct.

### 3.7 Structure modeling and processing

**Fig. 5:**
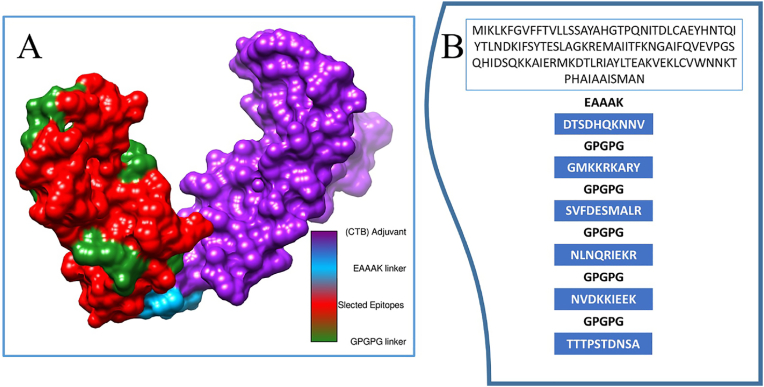
3D structural and schematic illustration of epitopes-based vaccine design. (A) 3D surface representation of the vaccine construct. Selected epitopes (red), GPGPG linkers (green), EAAAK linker (deep sky blue), and the cholera toxin B subunit (CTB) adjuvant is represented by purple color.

In structure modeling phase, the sequence of vaccine construct was submitted to Scratch Protein Predictor for structure modeling. The modeled structure was further visualized using the UCSF chimera v1.19. Fig. 5A illustrates the three-dimensional structure of the vaccine which comprised of an adjuvant molecule, linkers, and selected epitopes. Additionally, schematic depiction of the proposed vaccine is depicted in Fig. 5B. Moreover, the quality of the model structure was validated using the PDBsum Generate bio tool. The vaccine construct 90.2 % of the residues were plotted in the most favorable regions. S-Fig. 4 depicts the 3D, secondary structure and Ramachandran plot of vaccine construct.

### 3.8 Refinement of structure

The modeled structure was refined to enhance its structural stability by reconstructs side chains to achieve proper conformation. Comparatively, the predicted model 1 structure was found as the most refined considering the refinement score as described in S-Table 5. The modeled structure better plotting of the residues in Ramachandran favored regions and has less clash score compared to the reference.

### 3.9 Disulfide engineering and computational cloning of vaccine construct

**Fig. 6:**
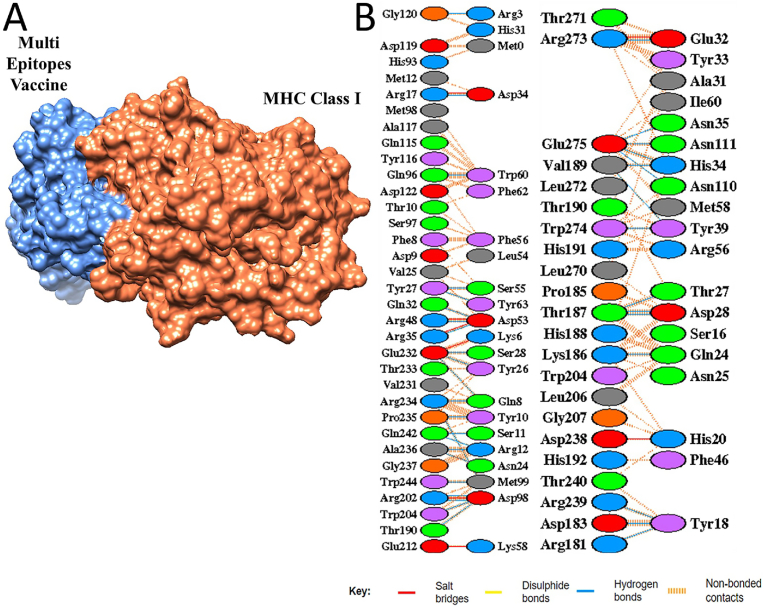
(A). The intermolecular docking binding mode between the vaccine candidate (blue) and the MHC-I (orange). **(B)** illustrates interaction map highlights key intermolecular forces and amino acid residues. The interactions include salt bridges (red), disulfide bonds (yellow), hydrogen bonds (blue), and non-bonded contacts (orange dashed lines).

The structural stability of the designed vaccine construct is essential. The refined vaccine was further stabilized through disulfide engineering. The position and geometric composition of amino acid residue pairs were analyzed to assess disulfide bond formation. The design 2.0 webserver predicted 19 pairs (Phe9-Ile26, Val12-Asn25, Leu13-Ala19, Gly21-Gln24, Cys30-His34, Thr36-Tyr39, Glu57-Ala67, Ala67-Gln82, Trp109-Lys112, Ala123-Ala128, Ala127-Thr131, Thr131-His134, Gly140-Gly144, Gly145-Ala166, Met146-Ala151, Gly158-Arg178, Arg182-Lys191, Asn'188-Asn209, Asp190-Ile193) for disulfide bond formation. These pairs of amino acid residues were replaced by cysteine amino acid to make the structure more stable. The original and mutated engineered vaccine structures are depicted in S-Fig. 5. The potential host expression system of vaccine construct was analyzed using computational cloning approach. Initially, the vaccine construct sequence was converted into a DNA sequence. The codon adaptation index value of the DNA sequence was 0.96 whereas the GC content value of 59.53 % was determined. The DNA sequence (ATGATCAAACTGAAATTTGGCGTCTTCTTCACCGTCCTGCTGTCTTCTGCTTACGCTCACGGTACCCCGCAGAACATCACCGACCTGTGCGCTGAATACCACAACACCCAGATCTACACCCTGAACGACAAAATCTTCTCTTACACCGAATCTCTGGCTGGTAAACGTGAAATGGCTATCATCACCTTCAAAAACGGTGCTATCTTCCAGGTTGAAGTTCCGGGTTCTCAGCACATCGACTCTCAGAAAAAAGCTATCGAACGTATGAAAGACACCCTGCGTATCGCTTACCTGACCGAAGCTAAAGTTGAAAAACTGTGCGTTTGGAACAACAAAACCCCGCACGCTATCGCTGCTATCTCTATGGCTAACGAAGCTGCTGCTAAAGACACCTCTGACCACCAGAAAAACAACGTTGGTCCGGGTCCGGGTGGTATGAAAAAACGTAAAGCTCGTTACGGTCCGGGTCCGGGTTCTGTTTTCGACGAATCTATGGCTCTGCGTGGTCCGGGTCCGGGTAACCTGAACCAGCGTATCGAAAAACGTGGTCCGGGTCCGGGTAACGTTGACAAAAAAATCGAAGAAAAAGGTCCGGGTCCGGGTACCACCACCCCGTCTACCGACAACTCTGCT) was cloned at the cleaved sites of pET-28a (+) vector using SnapGene Software. S-Fig. 6 illustrates the pET-28a (+) vector map.

### 3.10 Molecular docking analysis

**Fig .7:**
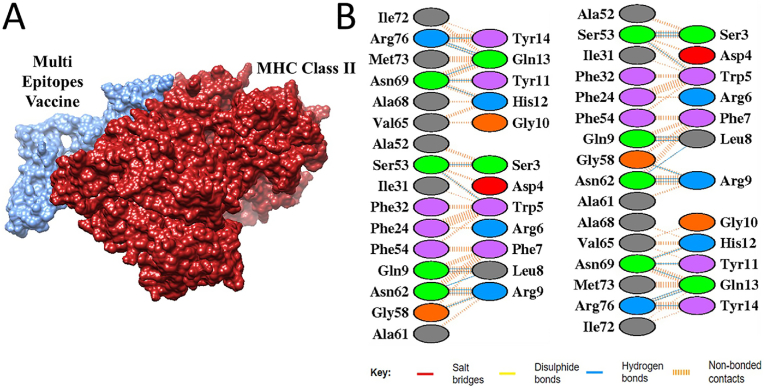
(A) Vaccine candidate (blue) binding to MHC-II (maroon). (B) illustrates intermolecular interactions map. The different interactions formed include salt bridges (red), disulfide bonds (yellow), hydrogen bonds (blue), and non-bonded contacts (orange dashed lines).

In molecular docking analysis, protein-protein docking was performed to examine the interactions between vaccine construct and immune cell receptors. The ClusPro server generated 10 docked complexes. The docked complexes were ranked by clustering properties, binding energy scores and size of the clusters. The docked complex in each receptor case with lowest binding energy score and largest cluster size (contain the most similar structural conformations) was selected for MD simulation analysis. The lowest binding energy complexes indicate their strong intermolecular affinity and stable binding conformation [53]. Further, the stable binding of vaccine construct, MHC-I and MHC-II molecules illustrates efficient presentation of the vaccine construct antigens to the host immune system [54]. The MHC-I-Vaccine complex cluster members and energy scores are presented in Table 2. The docking results of the MHC–II–Vaccine complex is presented in Table 3. Additionally, the docked complexes were visualized in UCSF Chimera v1.19 to evaluate the binding interactions. Salt bridges, disulfide bonds, and hydrogen bonds was predominantly seen. Fig. 6, Fig .7 represent the intermolecular docked conformation of MHC-I-Vaccine complex and MHC–II–Vaccine complex, respectively.

**Table 2:** The molecular docking result of MHC-I-Vaccine complex where binding poses are grouped into clusters. Each cluster includes the number of conformations, a representative pose (Center) with its corresponding weighted score and lowest energy pose. Lower binding energy scores specify stronger binding affinities.

Cluster	Members	Representative	Weighted Score
**0**	109	Center	−729.0
		Lowermost Energy	−833.0
**1**	72	Center	−801.9
		Lowermost Energy	−875.8
**2**	57	Center	−804.3
		Lowermost Energy	−915.6
**3**	52	Center	−786.9
		Lowermost Energy	−904.9
**4**	47	Center	−711.7
		Lowermost Energy	−800.8
**5**	33	Center	−769.4
		Lowermost Energy	−818.8
**6**	32	Center	−819.6
		Lowermost Energy	−819.6
**7**	31	Center	−807.5
		Lowermost Energy	−807.5
**8**	30	Center	−714.8
		Lowermost Energy	−825.1
**9**	25	Center	−708.6
		Lowermost Energy	−768.0
**10**	24	Center	−830.6
		Lowermost Energy	−958.8

**Table 3:** MHC–II–Vaccine complex docking findings. The cluster includes the number of conformations with corresponding weighted score and lowest energy pose. Lower binding energy scores specify stronger binding affinities.

Cluster	Members	Representative	Weighted Score
**0**	43	Center	−918.8
		Lowermost Energy	−1001.6
**1**	42	Center	−849.2
		Lowermost Energy	−1069.7
**2**	37	Center	−818.6
		Lowermost Energy	−1053.1
**3**	34	Center	−912.2
		Lowermost Energy	−985.9
**4**	32	Center	−989.1
		Lowermost Energy	−1030.4
**5**	31	Center	−863.9
		Lowermost Energy	−1014.7
**6**	27	Center	−852.9
		Lowermost Energy	−919.8
**7**	26	Center	−992.3
		Lowermost Energy	−1135.8
**8**	25	Center	−851.3
		Lowermost Energy	−917.6
**9**	24	Center	−815.1
		Lowermost Energy	−963.3
**10**	21	Center	−962.9
		Lowermost Energy	−962.9

### 3.11 MD simulation analysis

**Fig. 8:**
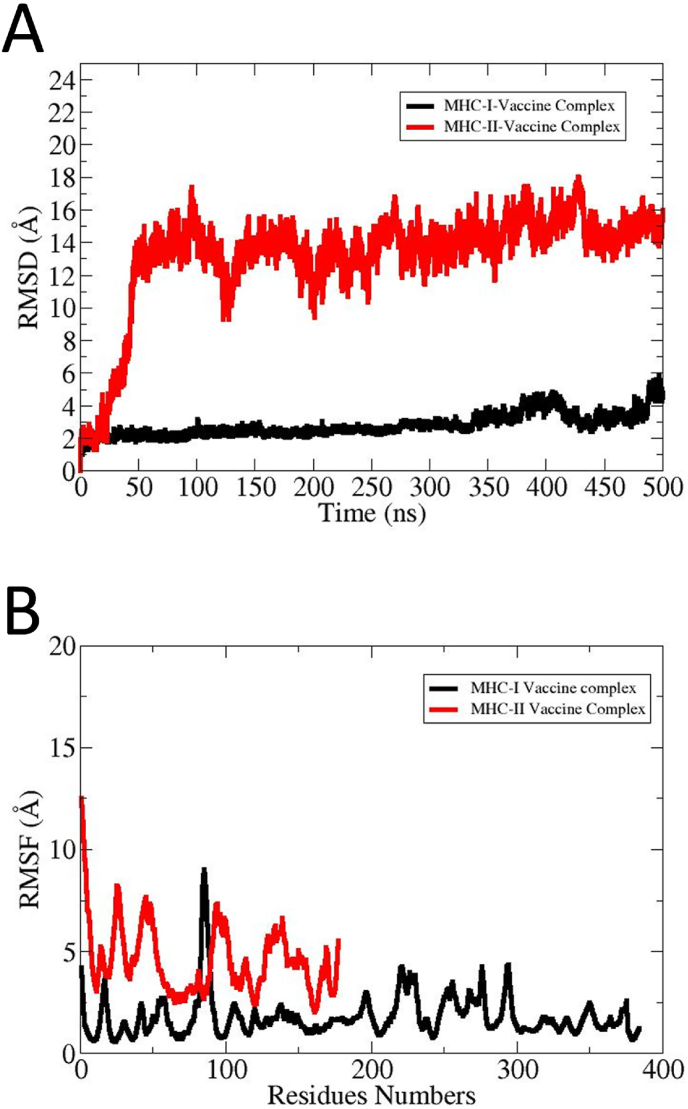
(A) The RMSD plot revealed structure dynamics of vaccine construct and receptors. The RMSD plots are based on carbon alpha atoms **(B)** The RMSF plot highlights the flexibility of individual residues and localized fluctuations. The residues level fluctuations were mostly seen in loop regions while core regions remain stable.

MD simulation was performed and RMSD (root mean square deviation), RMSF (root mean square fluctuation), and Rg (radius of gyration), SASA (solvent access surface area) and salt bridges plots were plotted for complexes. The simulation graphs provide valuable insights into the structural dynamic stability and flexibility of the vaccine and receptors. The RMSD graph illustrates that the MHC-I-Vaccine complex maintains stability with a lower RMSD across the simulation time demonstrating a higher structural stability as shown by black color Fig. 8A. The MHC–II–Vaccine complex is presented by red color in Fig. 8A. The MHC–II–Vaccine complex showed substantial fluctuations, which may be due to the high percentage of loops. Further, the binding groove of the MHC-II is in an open state and fluctuates more than the MHC-I, thus contributing to larger conformational changes [55]. However, the changes were found not to affect overall intermolecular MHC–II–Vaccine complex binding, and the vaccine's epitopes remained exposed to the host immune cells for immunological recognition and processing [56].Furthermore, the RMSF plot provides additional support for this observation where MHC-II complex showed higher fluctuations at specific regions, representing flexibility in the structure. The MHC-I-Vaccine complex remained comparatively stable across the simulation time. The RMSF simulation graphs of the MHC-I-Vaccine complex and MHC–II–Vaccine complex are presented by red and black in Fig. 8B.

### 3.12 Analysis of *Rg* and beta factor

**Fig. 9:**
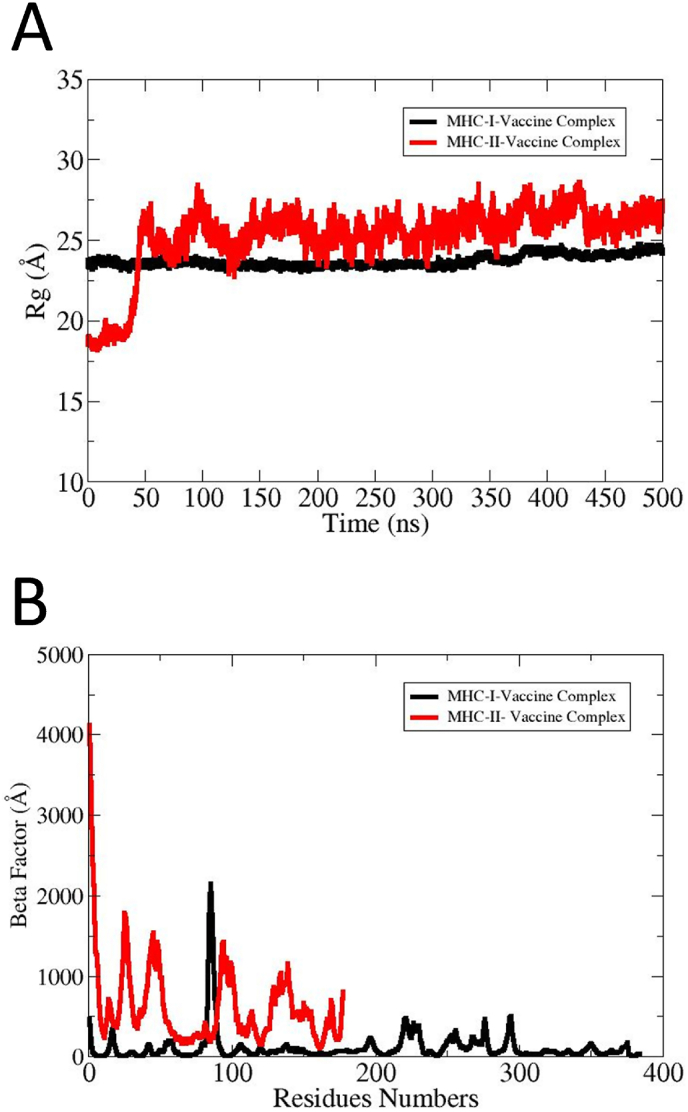
*Rg***(A)** and beta factor **(B)** plots of simulated complexes. The MHC–II–Vaccine complex (red) exhibits higher flexibility as compared to MHC-I-Vaccine complex (black).

The *Rg* analysis is a crucial analysis to evaluate structural compactness and relaxation of the simulated docked complexes. Also, it is vital to understand the dynamic interactions of docked molecules during the simulation time frame. The *Rg* plot represents that the MHC-I-Vaccine complex preserves a compact structure with negligible variation. In contrast, the MHC–II–Vaccine complex showed an increase in *Rg;* however, the complex system tends to achieve stability towards simulation end. Overall, findings of *Rg* propose that the MHC-I-Vaccine complex demonstrates greater structural stability while the MHC–II–Vaccine complex exhibits increased flexibility and dynamic behavior which may be due to high percentage of loops and open binding grooves. The *Rg* graph of MHC-I-Vaccine complex and MHC–II–Vaccine complex is presented in black and red color in Fig. 9A. The thermal presence atomic level fluctuations of the vaccine with MHC-I and MHC-II were analyzed by beta factor analysis. In beta-factor analysis, the MHC–II–Vaccine complex shows significantly higher beta factor values. The plot peak reaches above 4000 Å due to high conformational flexibility. The MHC-I-Vaccine complex shows comparatively low and stable beta-factor across the entire residues range. Several localized fluctuations were observed in the MHC–II–Vaccine complex for residues from 100 to 150, representing flexible loop regions. These fluctuations decline significantly beyond residue number 150, which depicts a relatively stable core domain. The minor fluctuation in beta-factor propose that the MHC-I-Vaccine complex is more stable and rigid demonstrating a strong binding interaction with fewer conformational changes. The beta-factor plots of MHC-I-Vaccine complex is shown by black color and MHC–II–Vaccine complex is shown by red color in Fig. 9.

### 3.13 SASA analysis

**Fig. 10:**
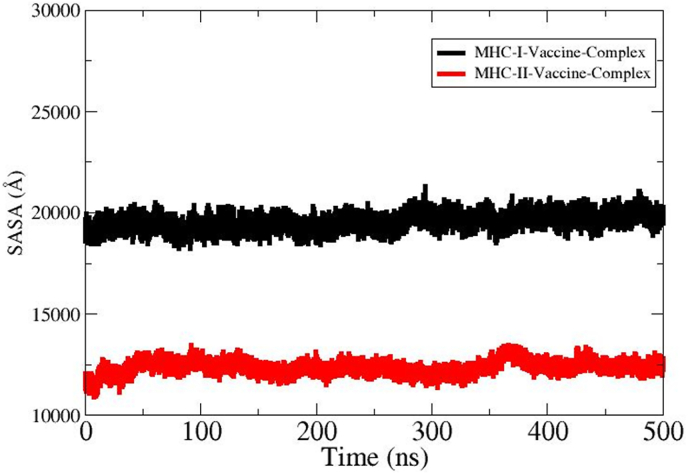
SASA plots of vaccine-immune receptors.

SASA is essential in evaluating the functional and structural characteristics of the multi-epitopes-based vaccine. SASA analysis primarily quantifies the exposed residues of proteins accessible to solvent molecules. This further provides mechanistic insights into vaccine complex folding, stability and overall interactions. Fig. 10 depicts SASA graphs of MHC-I-Vaccine complex and MHC–II–Vaccine complex. The MHC-I-Vaccine complex is mentioned in black color and MHC–II–Vaccine complex in red color. The SASA values for the vaccine MHC-I-Vaccine complex remain steadily higher around 20,000 to 22,000 Å compared to the MHC–II–Vaccine complex which stabilizes between 12,000 and 14,000 Å. This SASA analysis shows a greater solvent exposure for the MHC-I-Vaccine complex. The steady trends and slight fluctuations in SASA values during simulation time for both MHC-I-Vaccine complex and MHC–II–Vaccine complex suggest stable conformations. The SASA findings also reflects the robustness of the binding interactions between the vaccine and MHC-I and MHC-II receptors.

### 3.14 Hydrogen bonds (H-bonds) analysis

**Fig .11:**
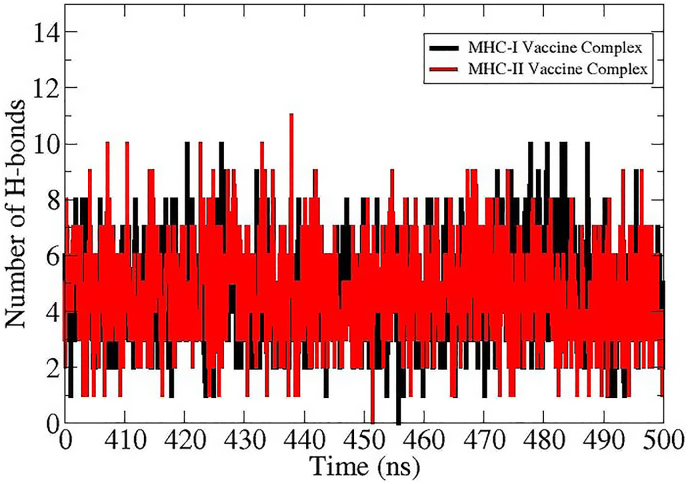
H-bonds analysis of MHC-I-Vaccine complex and MHC–II–Vaccine complexes.

H-bonds analysis is another important post-MD simulation assay. It delivers insights into the stability and strength of interactions. The H-Bonds graphs signify the number of H-bonds over the last 100 frames of 500 ns simulation time. Both the MHC-I-Vaccine complex and MHC–II–Vaccine complexes showed dynamic H-bonds formation. The MHC–II–Vaccine complex usually formed higher average number of H-bonds ranging from 4 to 8 as compared to MHC-I-Vaccine complex as it fluctuates between 2 and 6 H-bonds. The hydrogen bonds analysis indicates consistent interactions in MHC-I-Vaccine complex and MHC–II–Vaccine complexes. Further, comparatively higher H-bonds formation was observed in MHC–II–Vaccine complex which suggests stronger and more frequent polar interactions possibly contributing to improved binding stability of vaccine to MHC-II in its binding mechanism. In Fig. 11, the black and red color graph represents H-bonds of the MHC-I-Vaccine and MHC–II–Vaccine complex.

### 3.15 Salt bridges analysis

Salt bridges play a vital role as non-covalent interactions that improve stability and functional integrity of biomolecular systems. Salt bridge analysis mainly examined the dynamics of salt bridges across systems. Compared to MHC-I-Vaccine complex, the MHC–II–Vaccine complex displays a slightly reduced number of persistent salt bridges. The salt bridge analysis of the MHC-I-Vaccine complex and MHC–II–Vaccine complex delivers an understanding of their different electrostatic interactions and structural stability during simulation. In both MHC-I-Vaccine complex and MHC–II–Vaccine complexes, salt bridges formed and break dynamically. The blue color band represents the presence of salt bridges while red color represents the absence of salt bridge. The MHC-I-Vaccine complex displayed more stable and persistent salt bridges across multiple residue pairs representing stronger electrostatic interactions that contribute to its structural rigidity. In contrast, the MHC–II–Vaccine complex revealed few degrees of salt bridge disruption. It was observed that the MHC–II–Vaccine complex is more flexible and dynamic in nature. This aligns with the previous RMSD and Rg analysis, where the MHC–II–Vaccine complex showed some structural fluctuations. Stable salt bridges in the case MHC-I-Vaccine complex contribute to its compact and less fluctuating structure. While the transient interactions in MHC–II–Vaccine complex suggest a more adaptable but less rigid conformation, which could influence binding efficiency and vaccine stability. In the case of MHC-I-Vaccine complex; Asp220-Arg256, Glu180-Arg181, Glu350-Arg-373, Glu173lys176, Glu320-Arg321, Asp102-Arg6, Asp37-Arg35, Glu253-Lys37, Glu55-Arg170, Glu46-Arg35, Glu292-Lys295, Asp352-Lys317, Glu46-Arg35, Asp61-Arg62, Asp129-Arg131,Glu154-Arg151, Glu154-Arg131, Glu353-Arg373, Asp183-Arg239, Asp61-Arg44, Glu148arg151, Glu161-Arg131, Glu128-Arg111, Asp177-Arg181, Asp220-Arg256, Asp374-Arg202, Asp220-Arg256, Asp374-Arg202, Asp202-Arg256, Glu180-Arg181, Glu89-Arg82, Glu312-Arg357, Asp122-Lys121, Asp329-Arg48, Glu46-Arg44, Asp329-Arg48 amino acids pairs were observed in the salt bridges interaction. S-Fig. 7 depicts the salt bridges of MHC-I-Vaccine complex**.** The MHC–II–Vaccine complex shows a somewhat reduced number of persistent salt bridges and different amino acids pairs including Asp27-Arg42, Glu44-Arg48, Glu45-Arg48, Asp27-Arg42, Glu44-Arg48, Asp27-Arg42, Glu44-Arg48, Glu69-Lys73, Glu164-Arg162, Asp157-Arg121, Asp108-Arg138, Glu19-Lys145, Glu132-Lys145, Glu132-Lys145, Asp157-Arg121, Glu19-Lys145, Glu156-Arg98, Glu132-Lys145, Glu69-Lys65, And Asp108-Arg144 as can depicted in S-Fig. 8.

### 3.16 Relative free binding energy estimation of docked complexes

The MMGB/PBSA and MM/PBSA methods are popular post-simulation binding estimation methods. The methods are more accurate and closer to experimental results than docking. In MM/GBSA, binding energy was −277.98 kcal/mol and −290.48 kcal/mol for the MHC-I-Vaccine complex and MHC–II–Vaccine complex, respectively. In case of MM/PBSA, the net binding energy of MHC-I-Vaccine complex was −278.33 kcal/mol while −291.8 kcal/mol energy value for MHC–II–Vaccine complex was reported. These energy values interpret both complexes as strong intermolecular binders and produce robust interactions. The strongly bound MHC and vaccine molecules ensured continuous presentation of the vaccine antigens to the host immune system for antigenic processing, recognition, and generating protective immune responses. The different energies predicted for the MHC-Vaccine complexes are tabulated in S-Table 6. In one previous study, MM/PBSA approach was used to investigate a *Mycobacterium tuberculosis* subunit vaccine interaction energy for TLR-2 and TLR-3. The Vaccine-TLR2 complex and Vaccine-TLR3 complex reported a net MM/PBSA binding energy of −1480.96 kcal/mol and −1714.22 kcal/mol, respectively. [57]. In another study, a novel mRNA vaccine was engineered to combat *Acinetobacter baumannii* infections. In this study, the estimated MM/PBSA binding energy for Vaccine-TLR2 complex was −196.44 kcal/mol and −239.26 kcal/mol for Vaccine-TLR4 complex [58]. A multi-epitopes vaccine against *M. ulcerans* revealed MM/PBSA binding energy of −162.08 kcal/mol for TLR-2 and −142.00 for TLR-4 [59]. An immunoinformatics based multi-epitopes vaccine against *Leishmania donovani* unveiled MM/GBSA binding energy of −169.75 kcal/mol while the MMPBSA predicted was −1304 kcal/mol for TLR-4 receptor [60].

### 3.17 Host immune responses modeling evaluation (C-immune simulation)

**Fig .12:**
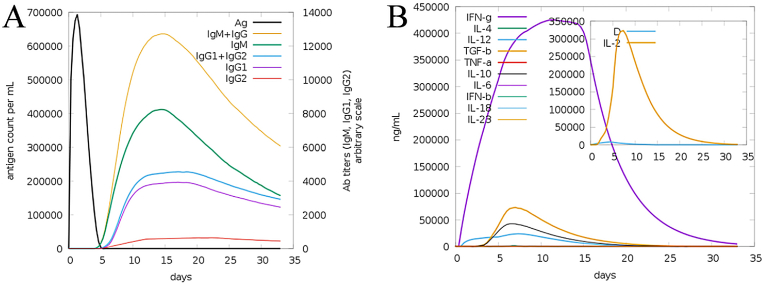
The in silico immune simulation of the host immune system against the vaccine antigen. It assesses immune system dynamics consisting of antigen recognition, immune cell activation, and molecular interactions offering an understanding into the predicted immune response against vaccine. **(A)** illustrates antibody production against the vaccine. **(B)** represents interferon gamma, interleukins and other types of cytokines.

The C-ImmSim results of the proposed vaccine design demonstrate a robust and lasting immunological response against *E. faecalis*. In Fig. 12A, the antigen counts as shown by the black peak immediately and rapidly decline demonstrating efficient immune recognition and clearance of the antigens. Secondly, the primary IgM response shown by the yellow peak is robust followed by a substantial increase in IgG1 and IgG2 antibody titers as shown by green and blue peaks signifying the development of prolonged antibody dependent enhancement (ADE) immunity. The predicted IgG and IgM titers against the antigen ranged from 600,000 to 650,000 antigen counts per milliliter. Moreover, in Fig. 12B different cytokines and interferons were observed including IFN-γ shown by a purple color peak. It displays a dominant peak representing a strong T helper 1 (Th1) cells mediated cellular immune response critical for pathogen clearance. The predicted stimulation level of interferon-gamma (IFN-γ) was 430,000 ng per milliliter. Additionally, other cytokines including interleukin-4 (IL-4) (represented by a yellow color) peak and interleukin-12 (IL-12) (shown by blue) also peak early supporting both Th1 and Th2 immune system activation. Additionally, IL-2 plays a significant role in T-cell proliferation during immune responses. The overall C- immune simulation results specify a stable immune response with strong antibody-mediated and cellular-mediated immunity supporting the potential efficacy of the proposed vaccine construct in producing long-lasting protection and eradication against *E. faecalis*. Though highly useful, the C-ImmSim simulation offers several limitations. The algorithm used for immune responses simulation against the vaccine antigen usually can't be translated into experimental testings. The algorithm employed can't differentiate much among different antigens etc. [61]. The C-ImmSim simulation predicted IgG and IgM antibody titers of approximately 750,000 antigen counts per milliliter in response to a multi-epitope vaccine against *A. baumannii*. Furthermore, an IFN-γ level of 450,000 ng/mL was observed between days 15 and 20 in response to the vaccine antigen [62]. Similarly, in another study, the C-ImmSim simulation predicted IgG and IgM antibody titers of approximately 650,000 antigen counts per milliliter against *S. pneumoniae* vaccine antigen [63]. The C-ImmSim server predicted humoral immune response exhibited a logarithmic growth phase from day three to seven, reaching a peak of approximately 600 cells per mm^3^ against SARS-CoV-2 virus [64]. In another work, the server predicted an IgG1 and IgG2 antibody titer of approximately 660,000 antigen counts per milliliter against a multi-epitopes vaccine against *E. faecium* [65].

## 4 Discussion

Recent developments in sequencing approaches have significantly accelerated genome sequencing of bacterial pathogens, making it much faster, therefore, thousands of newly sequenced complete genomes of bacterial pathogens are now available in sequencing databases, facilitating research studies such as the identification of novel vaccine candidates [66]. In this research, we selected 20 representative fully sequenced strains of *E. faecalis* for the multi-epitopes vaccine, considering the global threat of antibiotic resistance (AR) and the lack of any approved vaccine against *E. faecalis*. The entire protein dataset of the selected strains was initially analyzed using the subtractive proteomics framework. Subtractive proteomics is an in-silico filtration method employed to identify and select proteins of interest from large protein databases. In previous studies, the subtractive proteomics approach was applied in designing multi-epitopes vaccine construct against different pathogenic microorganisms [31,[67], [68], [69], [70]].

In the first phase of this approach, the core proteins were predicted from 20 strains of *E. faecalis*. Core proteins are essential for the pathogen's survival and are present among all strains of bacterial pathogens. These core protein sequences were considered for the identification of probable vaccine candidates. During the second phase, the core proteins underwent sub-cellular localization analysis. Subcellular localization analysis involves determining the specific location of a protein within a cell. In this analysis, only subcellular localized proteins such as extracellular membrane, outer membrane, and periplasmic membrane located proteins were considered for vaccine target identification [71]. Outer membrane proteins (OMPs) are crucial for keeping the integrity and selective permeability of the bacterial cell membrane and play a vital role in generating immune responses against bacterial pathogens. Genetic diversity among bacterial strains even within the same bacterial species has inhibited the development of vaccines [72].However, (OMPs) bacterial proteins are conserved across serotypes and might be used as possible vaccine candidates. Furthermore, virulent proteins are often studied as good vaccine candidate due to their critical role in the pathogenesis of the pathogen and involvement in pathological events [73]. In the third phase, virulent proteins were predicted using the VFDB database. To make it easier to choose appropriate vaccine targets for experimental study evaluation that can be quickly applied in vaccine development, the physicochemical properties of the pathogenic proteins were then assessed.

Virulent proteins were subsequently evaluated according to the defined cutoff value of <110 (kDa) molecular weight. Protein stability is an essential factor and should be considered during the selection of potential vaccine targets [74]. The instability index calculator determines the stability of protein by predicting the presence of specific dipeptides that are found in stable protein but absent in unstable protein in vivo. Since proteins with a computed value greater than 40 are classified as unstable, 40 was set as the threshold value for the protein instability index. Antigenicity of the proteins is the ability of the proteins to bind specifically to the products of adaptive immunity such as B-cell and T-cell receptors. Only antigenic proteins were selected and non-antigenic proteins were discarded. Furthermore, to avoid the allergic response of vaccine, the allergic proteins were discarded, and non-allergic proteins were selected for BLASTp analysis. All selected proteins exhibited no hits in BLASTp analysis against human, probiotic proteomes and reference mouse proteome. The mouse has been an ideal mammalian model for experimental research over the past century. Mouse models exhibit several genetic similarities to humans, thereby providing useful insights for the study of human diseases [75].

In order to eradicate or prevent the growth of pathogens, the host's innate and adaptive immune systems are highly specialized and systemic. Adaptive immunity generates memory immune cells that recognize infections during subsequent contacts after first detection. Vaccination is based on the immunological memory of acquired immunity [76]. The hosts innate and adaptive immune systems are highly specific and comprehensive in eliminating or inhibiting pathogen growth. Adaptive immunity primarily produces memory immune cells that identify pathogens on consequent encounters following first recognition. The adaptive memory of acquired immunity constitutes the basis of vaccination [77]. The key components of the adaptive host defense system are B-cell and T-cell, which account for producing antibody-mediated and cell-mediated immune responses, respectively. The selected proteins were analyzed for both B-cell and T-cell epitopes. The prediction of B-cell epitopes is essential as antibodies bind to epitopes and activate protective mechanisms, including (i) agglutination (a process that results in cell aggregation, facilitating pathogen recognition and elimination by the host organism) (ii) complement activation (for inflammation and cellular destruction), opsonization (a process of labeling foreign antigens to promote phagocytosis), neutralization (prevention of bacterial attachment to target mucosa) and T cell-mediated immune responses (cytotoxicity and macrophages targeting cells marked by antibodies) [78].Additionally, the anticipated B-cell epitopes underwent T-cell epitopes prediction, wherein MHC-I and MHC-II epitopes were predicted. MHC-I molecules are surfaced on nucleated cells (WBCs) and present epitopes derived from both intracellular and extracellular proteins via cross-presentation to cytotoxic T cell eliciting a rapid and robust immune response to eliminate the pathogen [79]. The predicted epitopes were subsequently prioritized according to the immunoinformatics filters as mentioned in methodology section **(2.3. Epitopes Prediction and Selection)**. The selected epitopes include continuous B-cell and T-cell epitopes aimed at stimulating both cellular and humoral defense system against pathogen. Additionally, only epitopes with the highest binding ability to the HLA-DRB1∗0101 were opted as this allele is the most prevalent and widely dispersed allele in the human population. The epitopes that interact with this allele have the potential to elicit robust immune responses [80].

Vaccines based on whole organisms or large proteins have shown promising results in eradicating infectious diseases [81]. However, an increased antigenic load in these vaccines may result in inaccurate consequences and hyperimmune responses. Epitope-based vaccine construct is an effective alternative by generating specific immune responses. Furthermore, epitope-based vaccine construct is easy to synthesize, economical, and suitable for experimental and clinical applications. Additionally, epitopes-based vaccines pose a low risk of antigen-induced anaphylaxis reaction [82]. An epitopes-based vaccine designing has low immunogenicity and requires an appropriate adjuvant. The use of an adjuvant with a multi-epitope's vaccine design can enhance the efficacy of the vaccine composition. In the vaccine design, GPGPG linkers were employed to facilitate epitopes presentation and processing to the immune systems. Moreover, these linkers retain the separation of epitopes and prevent folding. Following to the construction of the vaccine construct, it was connected to the cholera toxin B at the N-terminal using EAAAK linker. The CTB is a non-toxic protein with molecular weight of 55 (kDa) that is capable of generating high magnitude of immune responses against the antigen to which it is attached. Furthermore, it has a maximum tendency to bind to monosialotetrahexosylganglioside (GM1), which is present on neuronal cell membranes of mammalian cells (macrophages, dendritic cells, gut epithelial cells and antigen processing cells [83].

The engineered multi-epitopes vaccine construct underwent an investigation of its physicochemical properties. The vaccine candidate was found stable and has a smaller molecular weight of 22.862 kDa. The 3D structure of the vaccine was modeled and underwent quality assessment. This analysis predicted that 90.2 % of amino acid residues occurred in the most favorable regions of the Ramachandran plot, thus representing an ideal 3D model for further downward analyses. The structure was further refined based on several refinement parameters mentioned in S-Table 5. The model 1 was selected for further analysis. The structure refinement aimed to enhance the quality and stability of the 3D structure. This process is important for ensuring that the designed vaccine can successfully activate the immune system [84]. Disulfide engineering was conducted to improve conformational stability of designed vaccine construct by minimizing configurational entropy. Disulfide bonds have been investigated for both inter-chain and intra-chain connections. After disulfide engineering, sequence of the vaccine construct was reverse transcribed into DNA sequence and then cloned into pET-28a (+) to improve expression in the *E. coli* expression system [40]. In silico immune simulation presented a substantial rise in adaptive immune responses after vaccine antigen exposure demonstrated by elevated antibody titers of IgM and IgG production [85].

Molecular docking analysis offers several benefits such as assessment concerning binding potential of the vaccine with receptor proteins and structure insights. In the molecular docking phase, protein-protein docking analysis was conducted to explicate the best binding interaction mode of the vaccine to MHC-I and MHC-II molecules [86]. Blind docking analysis was done by exposing the whole surface of selected receptors to the vaccine for binding [44].The server produces 10 docked complexes. Through evaluation of clustering characteristics, lowest binding energy scores, and cluster sizes, the top docked complex was chosen [87]. Similar to our study, Emmanuel et al., 2023 uses docking analysis (by employing ClusPro 2.0 server) predicted potential epitopes from OmpB and heat shock protein GroEl against rickettsioses [53]. They selected the lowest energy solution (−1159.6 kcal/mol) among the 30 generated complexes as the best docked complex. Though docking is useful method for intermolecular binding prediction, it has several limitations including inaccuracies of the scoring function, sampling limitations, protein flexibility, shortcoming of force field used and validation challenges etc. [88]. The simulation graphs such as RMSDF and RMSF provide valuable insights into the structural dynamic stability and flexibility of the vaccine with MHC-I and MHC-II molecules. In RMSD and RMSF analysis, the vaccine-MHC-II shows minor fluctuations compared to MHC-I-Vaccine complex that may arise from the loops present in the protein structure or the open-ended binding groove of the MHC-II receptor. The loops are naturally flexible regions and upon complexes visualization, it was found that the intermolecular binding conformation and interaction remain strongly intact [89]. In a recent study by Anoop et al., 2024 used the MD method to confirm the binding stability of vaccine receptor complexes against NIPAH virus [90]. Further, the SASA analysis was done to assess solvent exposed surface area of the complex and its impact on intermolecular binding and interactions. The SASA plots suggested stable conformations for both complexes. This further indicates that due to less conformational deviations, fewer complexes areas are exposed to the water molecules hence less chances of disturbance to affect complex intermolecular binding conformation and affect overall binding. The calculation of binding free energy of vaccine and target receptors is essential for understanding local interactions enabling the user to assess each residue's contribution to the total binding energy of the system [91].

This comprehensive computational analysis reported that the proposed engineered multi-epitopes vaccine construct can provoke immunological responses against *E. faecalis*. Although a rigorous selection criterion was maintained, the experimental evaluation of the vaccine construct's immune protection ability remains necessary. Moreover, cross-validation of the vaccine interactions with MHC-I and MHC-II molecules is essential [92]. The optimal organization and spacing of epitopes and linkers relative to the selection of the best delivery route must need additional validation to achieve maximum immunogenicity of vaccine construct. In one previous study, subtractive proteomics, immunoinformatics analysis, docking, and MD simulation approaches were applied for screening protective epitopes and designing multi-epitopes vaccine construct against *Listeria monocytogenes* [93]. The reverse vaccinology method has been successfully applied in developing *Meningococcus B* (MenB) vaccine [94].

Also, for other bacterial pathogens such as *Staphylococcus aureus* [95], *Chlamydia* [96], *Streptococcus pneumoniae* [97], and Group A *Streptococcus* [98], multi epitopes vaccines have been designed through reverse vaccinology methods. Similarly, reverse vaccinology, immunoinformatics and biophysics approaches have been applied to designing of multi-epitopes vaccine against several bacterial viral, parasitic and cancer such as *Brugia malayi* [99], *Escherichia albertii* [100], SARS-CoV-2 [101], *Acinetobacter baumannii* [62], *Mycobacterium avium* subspecies paratuberculosis [102], Hepatitis C Virus [103], *Gardnerella vaginalis* [104] and human respiratory syncytial virus (hRSV) [105] and oncogenic KRAS [106]. Using the proposed vaccine construct against *E. faecalis*, experimental studies need to be conducted for validation. The validation process may include to evaluate the effect of immunization on bacterial load, serum IgG antibody titers, serum cytokine and interferon levels and histopathological assessments in Balb/c mice and non-mutant zebrafish [107].

## 5 Conclusion

Due to the rapid increase of antibiotic resistance in bacterial pathogens, developing an effective vaccine against antibiotic resistance *E. faecalis* is essential. In this research, reverse vaccinology, immunoinformatics, biophysics, and host immune simulation approaches were applied to design a chimeric multi-epitopes vaccine in defense to emerging *E. faecalis* infections. The designed vaccine construct comprised multiple antigenic B-cell and T-cell epitopes capable of evoking antibody and cellular-mediated immune responses. Molecular docking analysis unveiled that the vaccine and MHC-I and MHC-II have better interactions. In the MD simulation analysis, it was observed that the docked complexes have proper intermolecular conformational stability in a dynamic environment. Moreover, the C-immune simulation analysis demonstrated that the vaccine construct is capable of efficiently interacting with target immune cells and can provoke both humoral and cell-mediated immune responses against *E. faecalis*. The proposed in silico-based vaccine construct is a ready to be used by experimental vaccinologists for further laboratory and animal studies to validate its efficacy against *E. faecalis* infections*.* The predicted vaccine model will significantly accelerate vaccine development against the target pathogen.

## CRediT authorship contribution statement

**Asad Ullah:** Data curation, Investigation, Methodology, Writing – original draft, Writing – review & editing. **Sadiq Azam:** Data curation, Investigation, Methodology, Supervision, Writing – original draft, Writing – review & editing. **Sajjad Ahmad:** Conceptualization, Data curation, Investigation, Methodology, Project administration, Writing – original draft, Writing – review & editing. **Ibrar Khan:** Data curation, Investigation, Methodology, Validation, Writing – review & editing. **Dalia M. Alammari:** Data curation, Investigation, Methodology, Resources, Writing – review & editing. **Sumra Wajid Abassi:** Data curation, Formal analysis, Investigation, Methodology, Resources, Writing – review & editing. **Dong-Qing Wei:** Data curation, Project administration, Resources, Writing – review & editing. **Fahad M. Alshabrmi:** Data curation, Investigation, Methodology, Writing – review & editing. **Mohammad Abdullah Aljasir:** Data curation, Investigation, Methodology, Writing – original draft, Writing – review & editing. **Eid A. Alatawi:** Data curation, Investigation, Methodology, Writing – review & editing.

## Declaration of competing interest

The authors declare that they have no known competing financial interests or personal relationships that could have appeared to influence the work reported in this paper.

## Data Availability

Data will be made available on request.
