# Supplementary figures and images for: Towards precision epitopes based vaccine against Enterococcus faecalis by integrating vaccinomics, reverse vaccinology and biophysics approaches
Source: Biochem Biophys Rep. 2025 Jun 10;43:102082. doi: 10.1016/j.bbrep.2025.102082 (PMC12182314; doi:10.1016/j.bbrep.2025.102082)

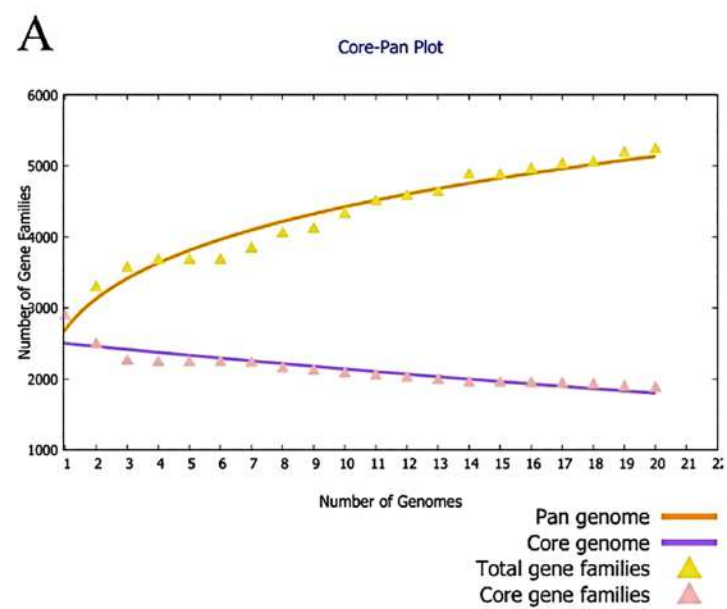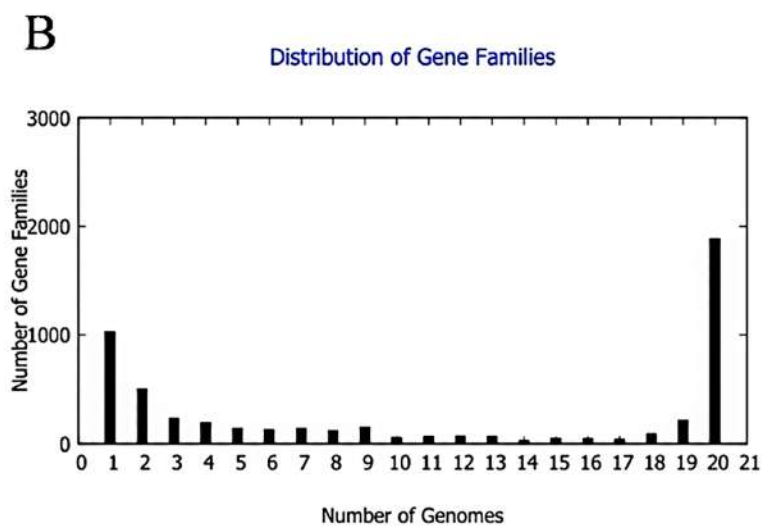

Supplement: Multimedia component 1 [file mmc1.pdf]

# Categories of Proteins

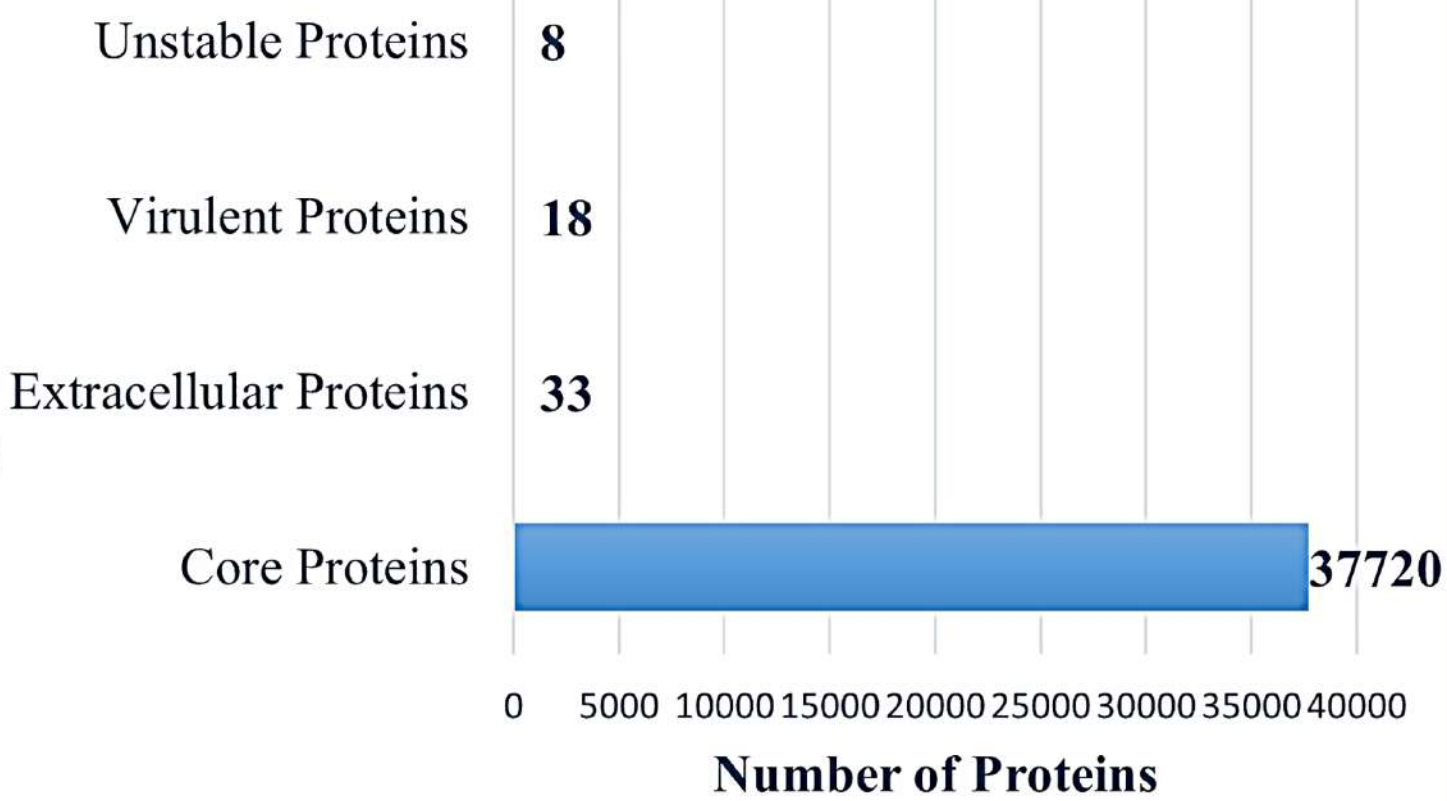

Supplement: Multimedia component 2 [file mmc2.pdf]

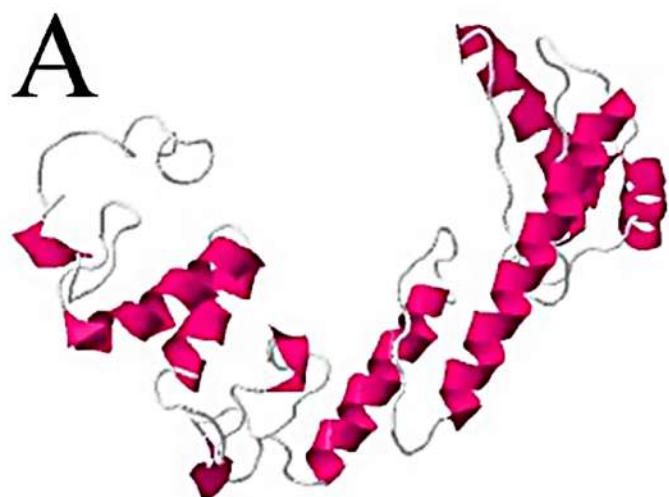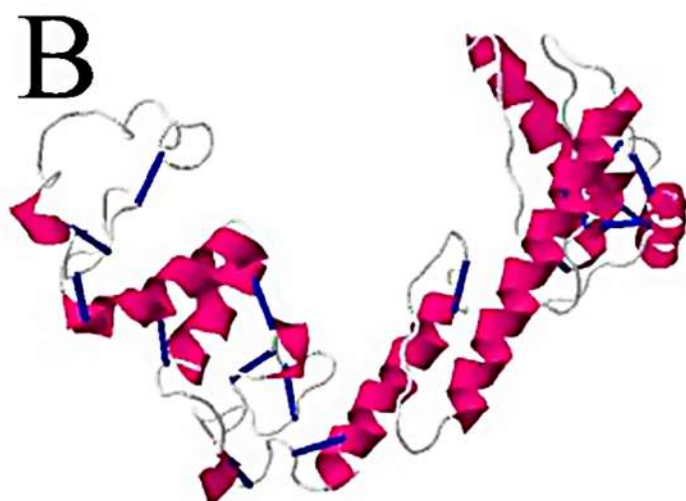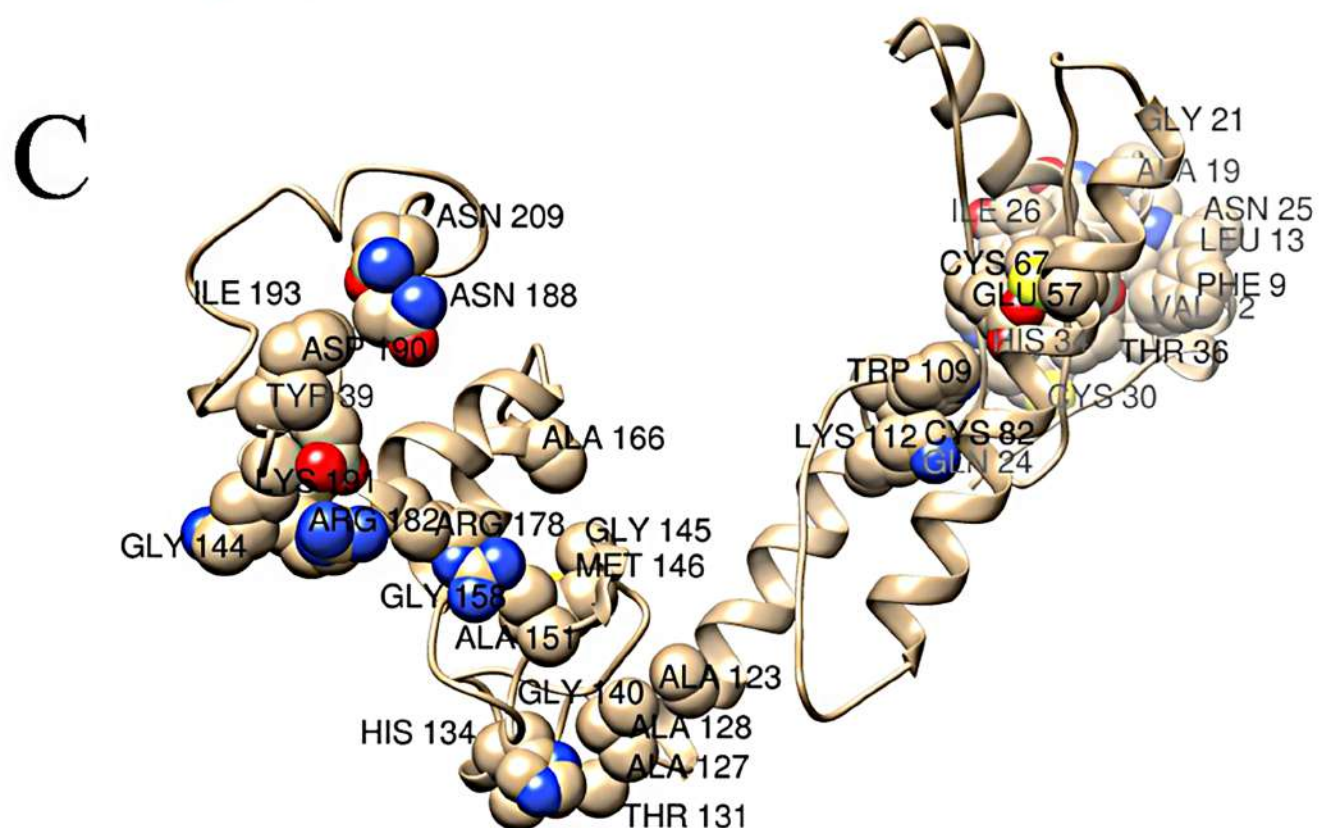

Supplement: Multimedia component 5 [file mmc5.pdf]
