# Supplementary material for: Towards precision epitopes based vaccine against Enterococcus faecalis by integrating vaccinomics, reverse vaccinology and biophysics approaches
Source: Biochem Biophys Rep. 2025 Jun 10;43:102082. doi: 10.1016/j.bbrep.2025.102082 (PMC12182314; doi:10.1016/j.bbrep.2025.102082)
Supplement: Multimedia component 3 [file mmc3.pdf]

A

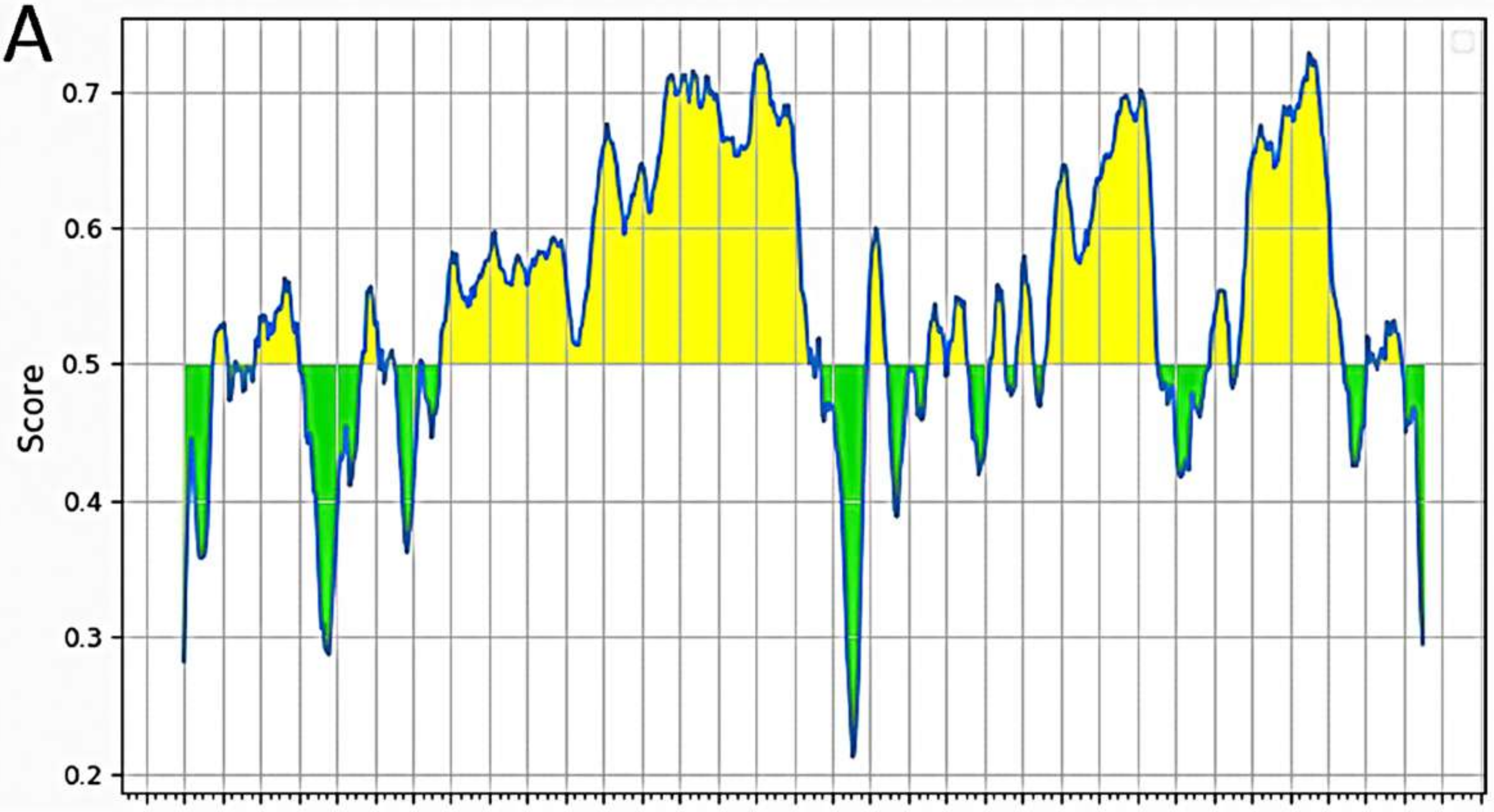

Query= core/3781/1/Org1_Gene1911
Glucosaminidase domain-containing protein

B

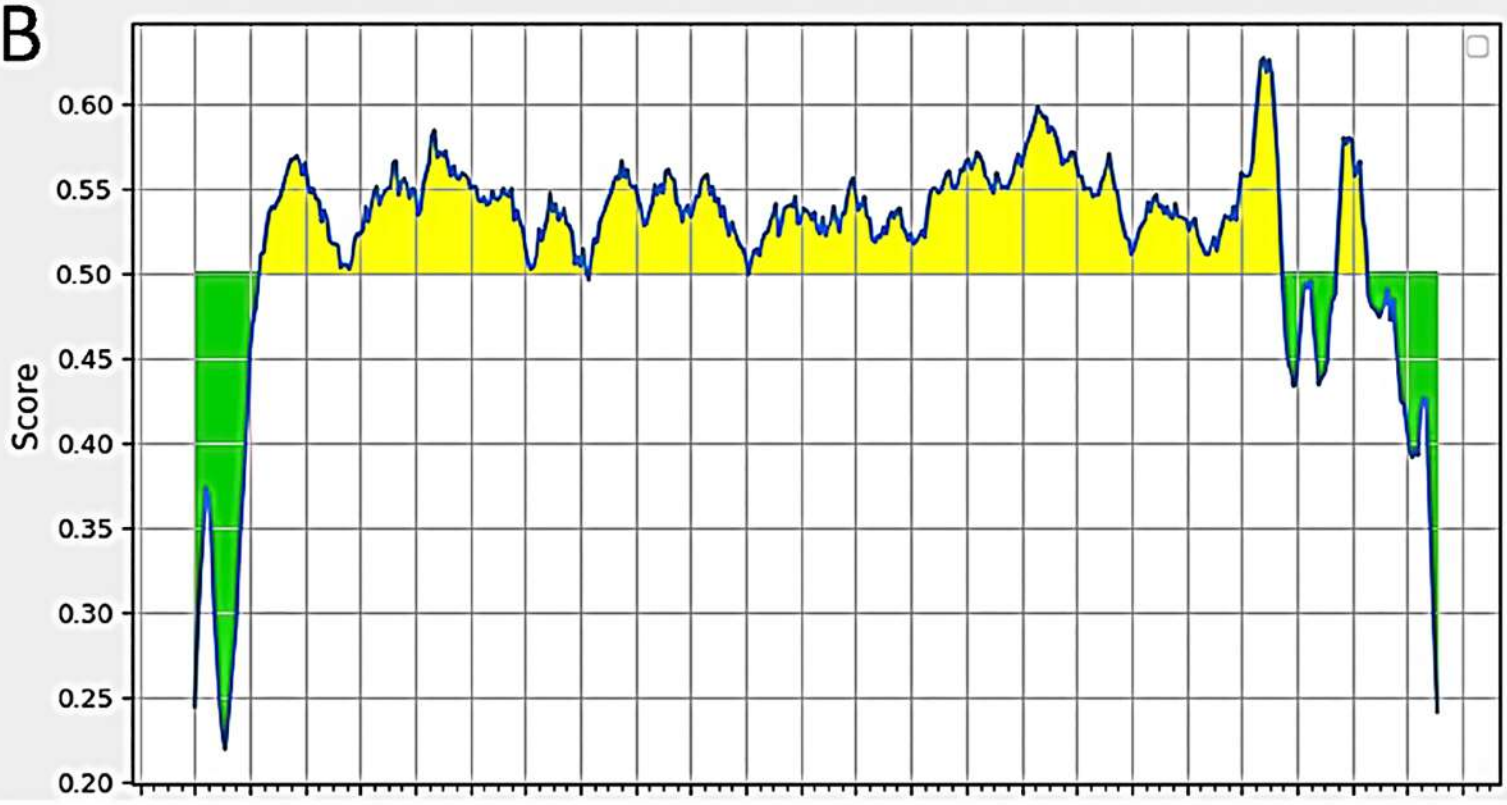

Query= core/845/8/Org8_Gene1547

Serine protease [*Enterococcus faecalis*]
