# Supplementary material for: Towards precision epitopes based vaccine against Enterococcus faecalis by integrating vaccinomics, reverse vaccinology and biophysics approaches
Source: Biochem Biophys Rep. 2025 Jun 10;43:102082. doi: 10.1016/j.bbrep.2025.102082 (PMC12182314; doi:10.1016/j.bbrep.2025.102082)
Supplement: Multimedia component 4 [file mmc4.pdf]

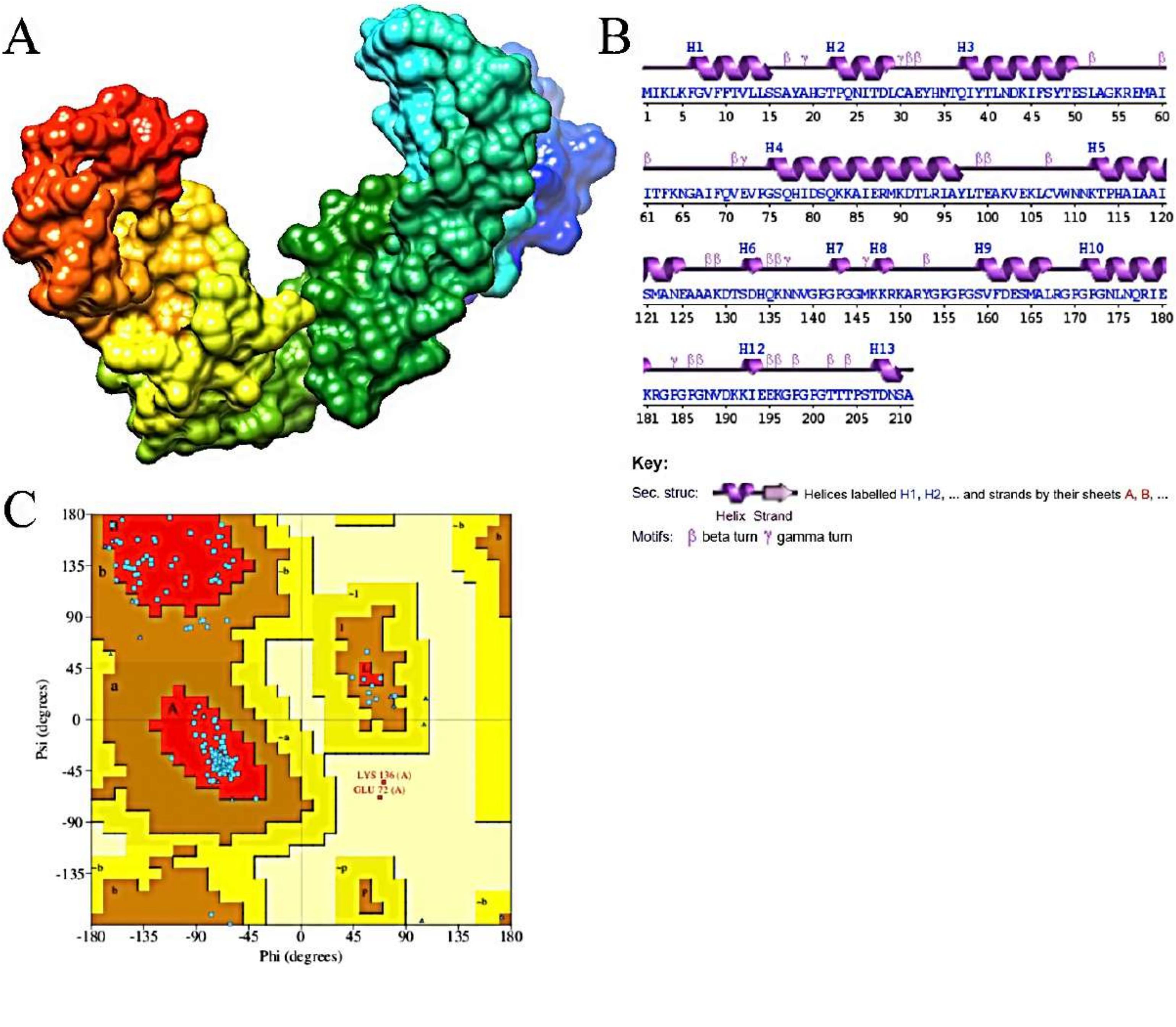
A
B
C
H1 H2 H3
MIKLKFGVFFTVLLSSAYAHGTPQNITDLCAEYHNTQIYTLNDKIFSYTESLAGKREMAI
1 5 10 15 20 25 30 35 40 45 50 55 60
H4 H5
ITFKNGAIFQVEVPGSQHIDSQKKAIERMKDTLRIAYLTEAKVEKLCVWNNKTPHAIAAI
61 65 70 75 80 85 90 95 100 105 110 115 120
H6 H7 H8 H9 H10
SMANEAAAKDTSDHQKNNVGFGFGGMKKRKARYGFGFGSVFDESMALRGFGFGNLNQRIE
121 125 130 135 140 145 150 155 160 165 170 175 180
H12 H13
KRGFGFGNVDKKIEEKGFGFGTTTPSTDNSA
181 185 190 195 200 205 210
Key:
Sec. struc: Helix Strand Helices labelled H1, H2, ... and strands by their sheets A, B, ...
Motifs: β beta turn γ gamma turn
Psi (degrees)
Phi (degrees)
LYS 136 (A)
GLU 72 (A)
