# Supplementary material for: Towards precision epitopes based vaccine against Enterococcus faecalis by integrating vaccinomics, reverse vaccinology and biophysics approaches
Source: Biochem Biophys Rep. 2025 Jun 10;43:102082. doi: 10.1016/j.bbrep.2025.102082 (PMC12182314; doi:10.1016/j.bbrep.2025.102082)
Supplement: Multimedia component 6 [file mmc6.pdf]

ATGATCAAACTGAAATTTGGCGTCTTCTTCACCGTCCTGCTGTCTTCTGCTTACGCTCACGGTACCCCGCAGAACATCACCGA
CCTGTGCGCTGAATACCACAACACCCAGATCTACACCCTGAACGACAAAATCTTCTCTTACACCGAATCTCTGGCTGGTAAAC
GTGAAATGGCTATCATCACCTTCAAAAACGGTGCTATCTTCCAGGTTGAAGTTCCGGGTTCTCAGCACATCGACTCTCAGAAA
AAAGCTATCGAACGTATGAAAGACACCCTGCGTATCGCTTACCTGACCGAAGCTAAAGTTGAAAAACTGTGCGTTTGGAACA
ACAAAACCCCGCACGCTATCGCTGCTATCTCTATGGCTAACGAAGCTGCTGCTAAAGACACCTCTGACCACCAGAAAAACAAC
GTTGGTCCGGGTCCGGGTGGTATGAAAAAACGTAAA GCTCGTTACGGTCCGGGTCCGGGTTCTGTTTTCGACGAATCTATGG
CTCTGCGTGGTCCGGGTCCGGGTAACCTGAACCAGCGTATCGAAAAACGTGGTCCGGGTCCGGGTAACGTTGACAAAAAAA
TCGAAGAAAAAGGTCCGGGTCCGGGTACCACCACCCCGTCTACCGACAACTCTGCT

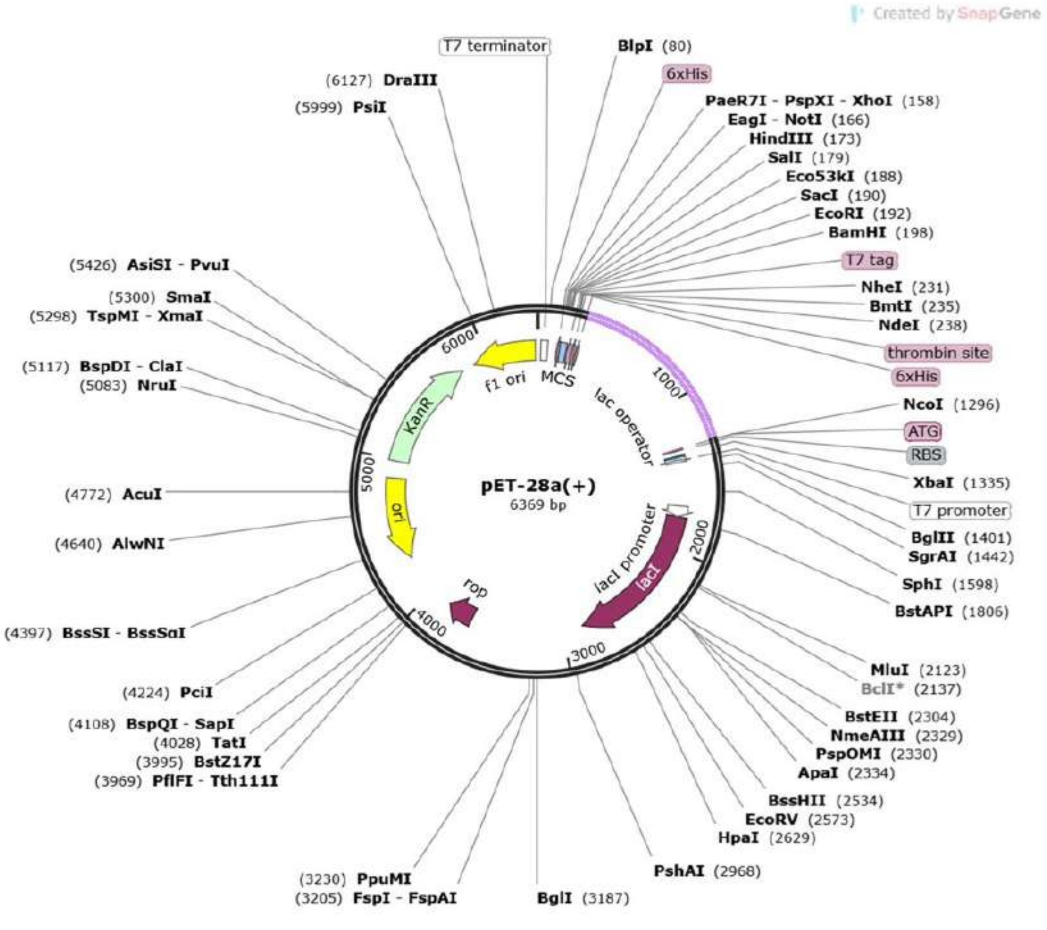

- CAI-Value of the improved DNA-sequence:0.966443
- GC-Content of the improved DNA-sequence:59.53%
