# Supplementary material for: Towards precision epitopes based vaccine against Enterococcus faecalis by integrating vaccinomics, reverse vaccinology and biophysics approaches
Source: Biochem Biophys Rep. 2025 Jun 10;43:102082. doi: 10.1016/j.bbrep.2025.102082 (PMC12182314; doi:10.1016/j.bbrep.2025.102082)
Supplement: Multimedia component 7 [file mmc7.pdf]

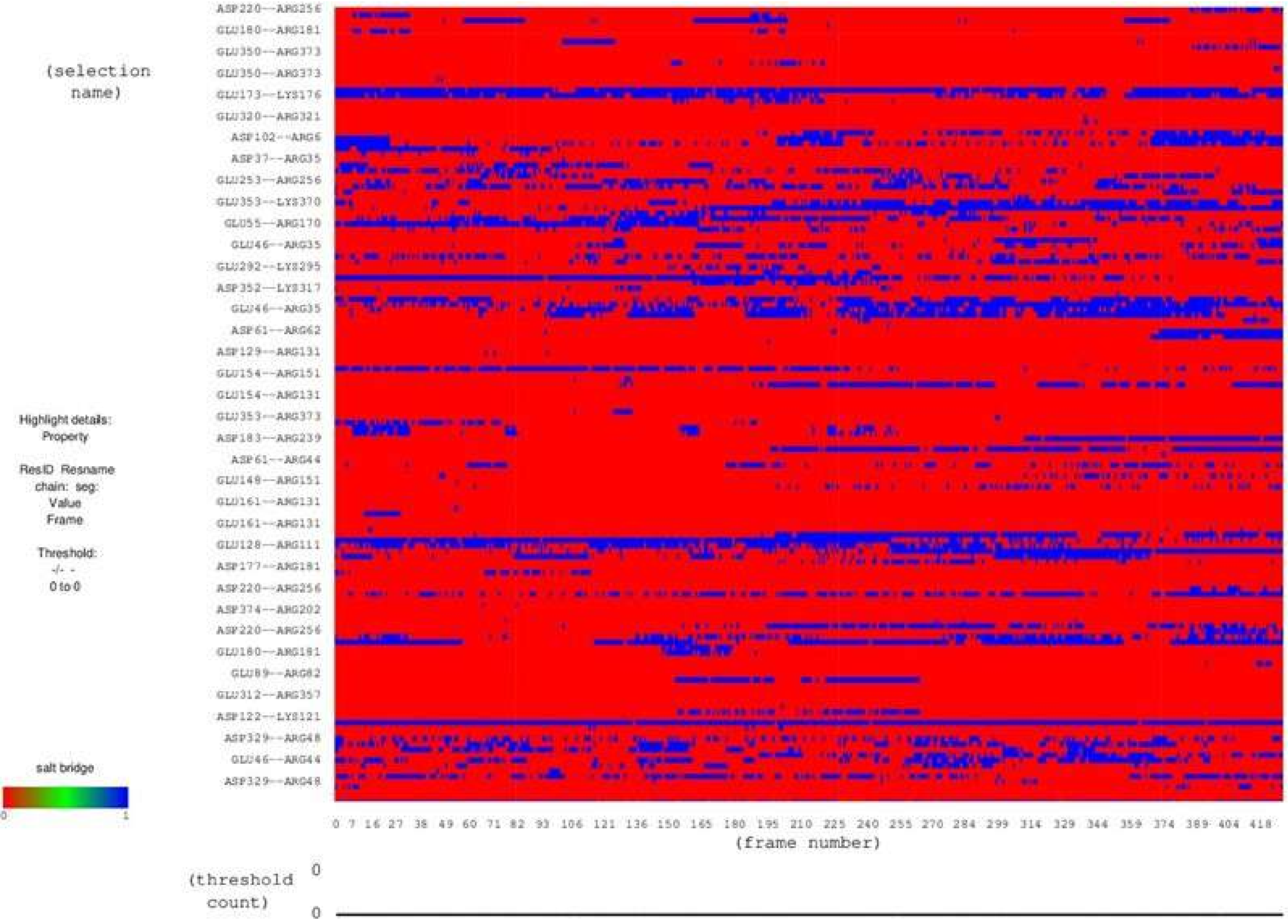
(selection name)
ASP220--ARG256
GLU180--ARG181
GLU350--ARG373
GLU350--ARG373
GLU173--LYS176
GLU320--ARG321
ASP102--ARG6
ASP37--ARG35
GLU253--ARG256
GLU353--LYS370
GLU55--ARG170
GLU46--ARG35
GLU292--LYS295
ASP352--LYS317
GLU46--ARG35
ASP61--ARG62
ASP129--ARG131
GLU154--ARG151
GLU154--ARG131
GLU353--ARG373
ASP183--ARG239
ASP61--ARG44
GLU149--ARG151
GLU161--ARG131
GLU161--ARG131
GLU128--ARG111
ASP177--ARG181
ASP220--ARG256
ASP374--ARG202
ASP220--ARG256
GLU180--ARG181
GLU89--ARG82
GLU312--ARG357
ASP122--LYS121
ASP329--ARG48
GLU46--ARG44
ASP329--ARG48
Highlight details:
Property
ResID Resname
chain: seg:
Value
Frame
Threshold:
-/- -
0 to 0
salt bridge
0
1
0 7 16 27 38 49 60 71 82 93 106 121 136 150 165 180 195 210 225 240 255 270 284 299 314 329 344 359 374 389 404 418
(frame number)
(threshold count)
0
0
