# Supplementary material for: Towards precision epitopes based vaccine against Enterococcus faecalis by integrating vaccinomics, reverse vaccinology and biophysics approaches
Source: Biochem Biophys Rep. 2025 Jun 10;43:102082. doi: 10.1016/j.bbrep.2025.102082 (PMC12182314; doi:10.1016/j.bbrep.2025.102082)
Supplement: Multimedia component 8 [file mmc8.pdf]

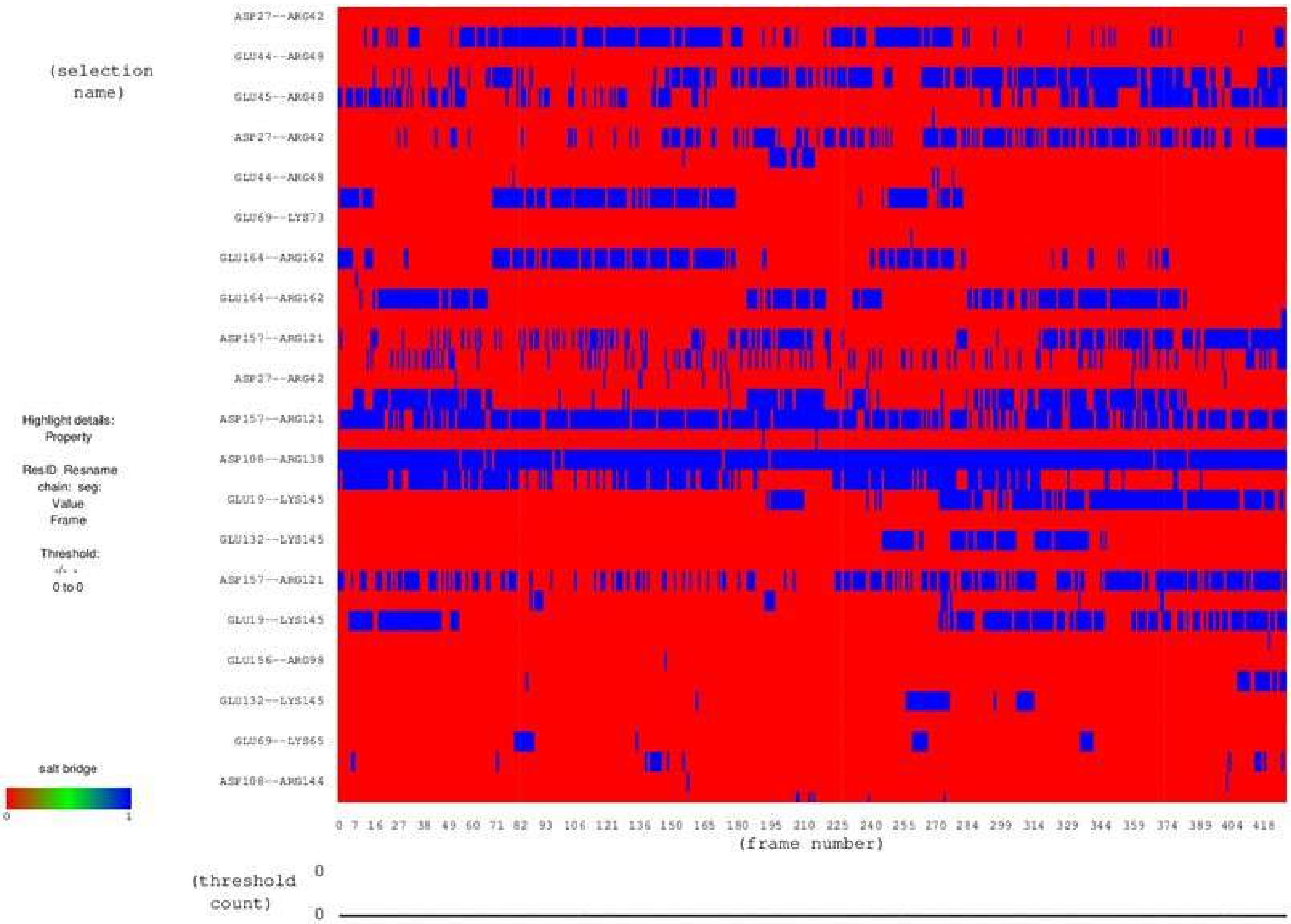
(selection name)
ASP27--ARG42
GLU44--ARG48
GLU45--ARG48
ASP27--ARG42
GLU44--ARG48
GLU69--LYS73
GLU164--ARG162
GLU164--ARG162
ASP157--ARG121
ASP27--ARG42
ASP157--ARG121
ASP108--ARG138
GLU19--LYS145
GLU132--LYS145
ASP157--ARG121
GLU19--LYS145
GLU156--ARG098
GLU132--LYS145
GLU69--LYS65
ASP108--ARG144
Highlight details:
Property
ResID Resname
chain: seg:
Value
Frame
Threshold:
-/- -
0 to 0
salt bridge
0
1
0 7 16 27 38 49 60 71 82 93 106 121 136 150 165 180 195 210 225 240 255 270 284 299 314 329 344 359 374 389 404 418
(frame number)
(threshold count)
0
0
